# Positive pressure therapy for Ménière’s disease

**DOI:** 10.1002/14651858.CD015248.pub2

**Published:** 2023-02-23

**Authors:** Katie E Webster, Ben George, Kevin Galbraith, Natasha A Harrington-Benton, Owen Judd, Diego Kaski, Otto R Maarsingh, Samuel MacKeith, Jaydip Ray, Vincent A Van Vugt, Martin J Burton

**Affiliations:** Cochrane ENT, Nuffield Department of Surgical SciencesUniversity of OxfordOxfordUK; Corpus Christi CollegeUniversity of OxfordOxfordUK; Cochrane ENTNuffield Department of Surgical Sciences, University of OxfordOxfordUK; Ménière’s SocietyWootonUK; ENT DepartmentUniversity Hospitals of Derby and Burton NHS Foundation TrustDerbyUK; National Hospital for Neurology and NeurosurgeryLondonUK; Department of General Practice, Amsterdam UMCVrije Universiteit Amsterdam, Amsterdam Public Health Research InstituteAmsterdamNetherlands; ENT DepartmentOxford University Hospitals NHS Foundation TrustOxfordUK; University of SheffieldSheffieldUK; Cochrane UKOxfordUK

**Keywords:** Adult, Humans, Meniere Disease, Meniere Disease/therapy, Otitis Media, Suppurative, Otitis Media, Suppurative/drug therapy, Physical Therapy Modalities, Tinnitus, Vertigo

## Abstract

**Background:**

Ménière's disease is a condition that causes recurrent episodes of vertigo, associated with hearing loss and tinnitus. It is often treated with medication, but different interventions are sometimes used. Positive pressure therapy is a treatment that creates small pressure pulses, generated by a pump that is attached to tubing placed in the ear canal. It is typically used for a few minutes, several times per day. The underlying cause of Ménière's disease is unknown, as is the way in which this treatment may work. The efficacy of this intervention at preventing vertigo attacks, and their associated symptoms, is currently unclear.

**Objectives:**

To evaluate the benefits and harms of positive pressure therapy versus placebo or no treatment in people with Ménière's disease.

**Search methods:**

The Cochrane ENT Information Specialist searched the Cochrane ENT Register; CENTRAL; Ovid MEDLINE; Ovid Embase; Web of Science; ClinicalTrials.gov; ICTRP and additional sources for published and unpublished trials. The date of the search was 14 September 2022.

**Selection criteria:**

We included randomised controlled trials (RCTs) and quasi‐RCTs in adults with a diagnosis of Ménière's disease comparing positive pressure therapy with either placebo or no treatment. We excluded studies with follow‐up of less than three months.

**Data collection and analysis:**

We used standard Cochrane methods. Our primary outcomes were: 1) improvement in vertigo (assessed as a dichotomous outcome ‐ improved or not improved), 2) change in vertigo (assessed as a continuous outcome, with a score on a numerical scale) and 3) serious adverse events. Our secondary outcomes were: 4) disease‐specific health‐related quality of life, 5) change in hearing, 6) change in tinnitus and 7) other adverse effects. We considered outcomes reported at three time points: 3 to < 6 months, 6 to ≤ 12 months and > 12 months. We used GRADE to assess the certainty of evidence for each outcome.

**Main results:**

We included three studies with a total of 238 participants, all of which compared positive pressure using the Meniett device to sham treatment. The duration of follow‐up was a maximum of four months.

**Improvement in vertigo**

A single study assessed whether participants had an improvement in the frequency of their vertigo whilst using positive pressure therapy, therefore we are unable to draw meaningful conclusions from the results.

**Change in vertigo**

Only one study reported on the change in vertigo symptoms using a global score (at 3 to < 6 months), so we are again unable to draw meaningful conclusions from the numerical results. All three studies reported on the change in the frequency of vertigo. The summary effect showed that people receiving positive pressure therapy had, on average, 0.84 fewer days per month affected by vertigo (95% confidence interval from 2.12 days fewer to 0.45 days more; 3 studies; 202 participants). However, the evidence on the change in vertigo frequency was of very low certainty, therefore there is great uncertainty in this estimate.

**Serious adverse events**

None of the included studies provided information on the number of people who experienced serious adverse events. It is unclear whether this is because no adverse events occurred, or whether they were not assessed and reported.

**Authors' conclusions:**

The evidence for positive pressure therapy for Ménière's disease is very uncertain. There are few RCTs that compare this intervention to placebo or no treatment, and the evidence that is currently available from these studies is of low or very low certainty. This means that we have very low confidence that the effects reported are accurate estimates of the true effect of these interventions. Consensus on the appropriate outcomes to measure in studies of Ménière's disease is needed (i.e. a core outcome set) in order to guide future studies in this area and enable meta‐analyses of the results. This must include appropriate consideration of the potential harms of treatment, as well as the benefits.

## Plain language summary

**What are the benefits and risks of positive pressure therapy for Ménière's disease?**

**Key messages**

Due to a lack of robust evidence, it is not clear whether positive pressure therapy works to improve the symptoms of people with Ménière's disease. We did not find any information on whether this treatment may cause any harm.

Larger, well‐conducted studies are needed in order to identify whether positive pressure therapy may be effective, and make sure that there are no harmful effects of treatment.

Further work also needs to be done to find out how best to measure the symptoms of people with Ménière's disease, in order to assess whether treatments are beneficial or not. This should include the development of a 'core outcome set' ‐ a list of things that should be measured in all studies on Ménière's disease.

**What is Ménière's disease?**

Ménière's disease is a condition that affects the inner ear. It causes repeated attacks of dizziness or vertigo (a spinning sensation), together with hearing problems, tinnitus (ringing, humming or buzzing noises in the ears) and a feeling of fullness or pressure in the ear. It usually affects adults, and starts in middle age.

**How is Ménière's disease treated?**

Oral medications (tablets) and lifestyle interventions are often used as the first treatment for Ménière's disease. If these do not work to control the symptoms, then other treatments may be tried. This includes positive pressure therapy, which involves placing a tube into the ear that is attached to a pump. The pump generates waves of pressure, which may help to reduce some symptoms of Ménière's disease. Typically, people use the pump for about five minutes at a time, up to three times per day.

**What did we want to find out?**

We wanted to find out:

‐ whether there was evidence that positive pressure therapy works to reduce the symptoms of Ménière's disease;

‐ whether positive pressure therapy might cause any harm.

**What did we do?**

We searched for studies that compared positive pressure therapy to either no treatment or sham (placebo) treatment.

**What did we find?**

We found three studies, which included a total of 238 people. They lasted for a maximum of four months, so we do not have any information about longer‐term effects of this treatment.

It is unclear whether positive pressure therapy makes any difference to symptoms of vertigo in the short term (up to four months).

We do not have any information on potential harms of the treatment (serious adverse effects) as none of the studies reported this.

**What are the limitations of the evidence?**

We have very little confidence in the evidence because the studies conducted were very small and had problems in their conduct, which means that the results may be unreliable. Larger, well‐conducted studies are needed to try and work out whether positive pressure therapy is effective.

**How up‐to‐date is this evidence?**

This evidence is up‐to‐date to September 2022.

## Summary of findings

**Summary of findings 1:** Positive pressure therapy compared to placebo for Ménière’s disease

**Positive pressure therapy compared to placebo for Ménière’s disease**						
**Patient or population:** adults with Ménière’s disease **Setting:** outpatients **Intervention:** positive pressure therapy **Comparison:** placebo						
**Outcomes**	Anticipated absolute effects^*^ (95% CI)		**Relative effect (95% CI)**	**№ of participants (studies)**	**Certainty of the evidence (GRADE)**	**Comments**
	**Risk with placebo**	**Risk with positive pressure therapy compared to no intervention/placebo**				
Improvement in vertigo frequency Follow‐up: range 3 months to 6 months	Study population		RR 1.19 (0.82 to 1.71)	77 (1 RCT)	⊕⊕⊝⊝ Low^1,2^	Positive pressure therapy may slightly increase the proportion of people in whom the frequency of vertigo improves at 3 to < 6 months.
	556 participants per 1000 would report that their vertigo frequency had improved	661 participants per 1000 would report that their vertigo frequency had improved (from 456 to 950 per 1000)				
Change in vertigo (global score) Assessed with: cumulative vertigo score in a 4‐week period (scale from: 0 to 112, higher scores = worse symptoms) Follow‐up: range 3 months to < 6 months	The mean change in vertigo (global score) was ‐1.19 points	MD 5.31 points lower (11.67 lower to 1.05 higher)	—	68 (1 RCT)	⊕⊝⊝⊝ Very low^2,3,4^	The evidence on the change in vertigo with positive pressure therapy is very uncertain (as measured with a global score at 3 to < 6 months).
Change in frequency of vertigo Assessed with: number of days with definitive vertigo episodes Follow‐up: range 3 months to < 6 months	The mean change in frequency of vertigo ranged from ‐0.42 to ‐4 days per month	MD 0.84 days per month lower (2.12 lower to 0.45 higher)	—	202 (3 RCTs)	⊕⊝⊝⊝ Very low^2,5^	The evidence on the change in vertigo frequency with positive pressure therapy is very uncertain at 3 to < 6 months.
Serious adverse events	No study reported this outcome					
*The risk in the intervention group (and its 95% confidence interval) is based on the assumed risk in the comparison group and the **relative effect** of the intervention (and its 95% CI). **CI:** confidence interval; **MD:** mean difference; **RCT:** randomised controlled trial; **RR:** risk ratio						
**GRADE Working Group grades of evidence** **High certainty:** we are very confident that the true effect lies close to that of the estimate of the effect. **Moderate certainty:** we are moderately confident in the effect estimate: the true effect is likely to be close to the estimate of the effect, but there is a possibility that it is substantially different. **Low certainty:** our confidence in the effect estimate is limited: the true effect may be substantially different from the estimate of the effect. **Very low certainty:** we have very little confidence in the effect estimate: the true effect is likely to be substantially different from the estimate of effect.						

^1^Risk of attrition bias and selective reporting. ^2^Sample size fails to meet optimal information size, taken as < 400 participants for a continuous outcome, or < 300 events for a dichotomous outcome, as a rule of thumb. ^3^Serious risk of attrition bias. ^4^Risk of bias due to use of an unvalidated scale for this outcome. ^5^Serious risk of attrition bias. Some concerns over selective reporting and other bias.

## Background

### Description of the condition

Ménière's disease was first described by Prosper Ménière in 1861 as a condition characterised by episodes of vertigo, associated with hearing loss and tinnitus (Baloh 2001). Sufferers may also report a feeling of fullness in the affected ear. Typically, it initially affects one ear, although some individuals may progress to develop bilateral disease. A hallmark of the condition is that symptoms are intermittent ‐ occurring as discrete attacks that last from minutes to several hours, then resolve. However, over time there is usually a gradual deterioration in hearing, and there may be progressive loss of balance function, leading to chronic dizziness or vertigo.

The diagnosis of Ménière's disease is challenging, due to the episodic nature of the condition, clinical heterogeneity, and the lack of a 'gold standard' diagnostic test. Even the agreed, international classification system has scope for two categories of diagnosis – 'definite’ and 'probable' (Lopez‐Escamez 2015). In brief, a diagnosis of definite Ménière's disease requires at least two episodes of vertigo, each lasting 20 minutes to 12 hours, together with audiometrically confirmed hearing loss and fluctuating aural symptoms (reduction in hearing, tinnitus or fullness) in the affected ear. 'Probable' Ménière's disease includes similar features, but without the requirement for audiometry to diagnose hearing loss, and with scope for the vertigo episodes to last longer (up to 24 hours). Both categories ('definite' and 'probable') require that the symptoms are not more likely to be due to an alternative diagnosis, due to the recognised challenges in distinguishing between balance disorders. 

Given the difficulties in diagnosis, the true incidence and prevalence of the disease are difficult to ascertain. A population‐based study in the UK using general practice data estimated the incidence to be 13.1 per 100,000 person‐years (Bruderer 2017), and the prevalence of the disease has been estimated at 190 per 100,000 people in the US (Harris 2010). It is a disorder of mid‐life, with diagnosis typically occurring between the ages of 30 and 60 (Harcourt 2014). Some studies report a slight female preponderance, and there may be a familial association, with approximately 10% of patients reporting the presence of the disease in a first, second or third degree relative (Requena 2014).

The underlying cause of Ménière's disease is usually unknown. Ménière's disease has been associated with an increase in the volume of fluid in the inner ear (endolymphatic hydrops). This may be caused by the abnormal production or resorption of endolymph (Hallpike 1938; Yamakawa 1938). However, it is not clear whether this is the underlying cause of the condition, or merely associated with the disease. Some authors have proposed other underlying causes for Ménière's disease, including viral infections (Gacek 2009), allergic (Banks 2012) or autoimmune disease processes (Greco 2012). A genetic predisposition has also been noted (Chiarella 2015). Occasionally, the symptoms may be secondary to a known cause (such as a head injury or other inner ear disorder) – in these cases it may be referred to as Ménière's syndrome.

Although Ménière's disease is relatively uncommon, it has a profound impact on quality of life. The unpredictable, episodic nature of the condition and severe, disabling attacks of vertigo cause a huge amount of distress. Quality of life (including physical and psychosocial aspects) is significantly reduced for those with Ménière's disease (Söderman 2002). The costs of the condition are also considerable, both in relation to medical interventions (appointments, diagnostic tests and treatments) and loss of productivity or sick days for those affected by the condition (Tyrrell 2016).

### Description of the intervention

A variety of different interventions have been proposed to treat people with Ménière's disease. These include dietary or lifestyle changes, oral treatments, treatments administered by injection into the ear (intratympanic) and surgical treatments. This review focuses on the use of positive pressure therapy to treat the symptoms of Ménière's disease.

Small pressure pulses are generated by a pump that is attached to tubing placed in the ear canal. Positive pressure therapy also requires the placement of a ventilation tube in the tympanic membrane, to encourage the pulsed pressure to transmit to the inner ear. The treatment is typically used for approximately five minutes at a time, and up to three times per day. 

At present, there is no agreement on which is the ideal treatment for people with Ménière's disease – consequently there is no 'gold standard' treatment with which to compare this intervention. 

### How the intervention might work

As the underlying cause of Ménière's disease is poorly understood, so too are the ways in which the interventions may work. 

Pressure changes in the middle ear can transmit to the inner ear through the round and oval windows. An increase in inner ear pressure has been suggested to result in decongestion of the inner ear vasculature, or to promote the opening of a temporarily blocked endolymphatic duct (Tjernström 1977). In this way, endolymph pressure is suggested to be altered. Intermittent positive pressure treatment was shown to reduce the development of endolymphatic hydrops in a guinea pig model (Sakikawa 1997). 

### Why it is important to do this review

Balance disorders can be difficult to diagnose and treat. There are few specific diagnostic tests, a variety of related disorders with similar symptoms, and a limited number of interventions that are known to be effective. To determine which topics within this area should be addressed with new or updated systematic reviews we conducted a scoping and prioritisation process, involving stakeholders (https://ent.cochrane.org/balance-disorders-ent). Ménière's disease was ranked as one of the highest priority topics during this process (along with vestibular migraine and persistent postural perceptual dizziness). 

Although Ménière's disease is a relatively uncommon condition, the significant impact it has on quality of life demonstrates the clear importance of identifying effective interventions to alleviate the symptoms. There is considerable variation in the management of Ménière's disease on both a national and international scale, with a lack of consensus about appropriate first‐line and subsequent therapies. 

This review is part of a suite of six that consider different interventions for Ménière's disease. Through these reviews, we hope to provide a thorough summary of the efficacy (benefits and harms) of the different treatment options, to support people with Ménière's (and healthcare professionals) when making decisions about their care. 

## Objectives

To evaluate the benefits and harms of positive pressure therapy versus placebo or no treatment in people with Ménière's disease.

## Methods

### Criteria for considering studies for this review

#### Types of studies

We included randomised controlled trials (RCTs) and quasi‐randomised trials (where trials were designed as RCTs, but the sequence generation for allocation of treatment used methods such as alternate allocation, birth dates etc). 

Ménière's disease is known to fluctuate over time, which may mean that cross‐over trials are not an appropriate study design for this condition. However, no cross‐over RCTs or cluster‐RCTs were identified as relevant for inclusion in this review.

We included studies reported as full‐text, those published as conference abstracts only and unpublished data. 

Ménière's disease is characterised by episodic balance disturbance ‐ the frequency of attacks may change over time (Huppert 2010). For studies to obtain accurate estimates of the effect of different interventions, we considered that follow‐up of participants should be for at least three months ‐ to ensure that participants are likely to have experienced a number of attacks during the follow‐up period. Studies that followed up participants for less than three months were excluded from the review.

#### Types of participants

We included studies that recruited adult participants (aged 18 years or older) with a diagnosis of definite or probable Ménière's disease, according to the agreed criteria of the American Academy Otolaryngology ‐ Head and Neck Surgery (AAO‐HNS), the Japan Society for Equilibrium Research, the European Academy of Otology and Neurotology and the Bárány Society. These criteria are outlined in Appendix 1 and described in Lopez‐Escamez 2015. 

If studies used different criteria to diagnose Ménière's disease, we included them if those criteria were clearly analogous to those described in Lopez‐Escamez 2015. For example, studies that used earlier definitions of Ménière's disease (from the AAO‐HNS guidelines of 1995) were also included. 

We anticipated that most studies would include participants with active Ménière's disease. We did not exclude studies if the frequency of attacks at baseline was not reported or was unclear, but we planned to highlight if there were differences between studies that may impact on our ability to pool the data, or affect the applicability of our findings.

We excluded studies where participants had previously undergone destructive/ablative treatment for Ménière's disease in the affected ear (such as vestibular neurectomy, chemical or surgical labyrinthectomy), as we considered that they were unlikely to respond to interventions in the same way as those who had not undergone such treatment.

#### Types of interventions

We included the following intervention:

- positive pressure therapy.

The main comparison is:

- positive pressure therapy versus placebo/no treatment.

##### Concurrent treatments

There were no limits on the type of concurrent treatments used, providing these were used equally in each arm of the study. We pooled studies that included concurrent treatments with those where participants did not receive concurrent treatment. We planned to conduct subgroup analysis to determine whether the effect estimates may be different in those receiving additional treatment. However, due to the small number of studies included in the review this was not possible (see Subgroup analysis and investigation of heterogeneity). 

#### Types of outcome measures

We assessed outcomes at the following time points: 

- 3 to < 6 months;
- 6 to ≤ 12 months;
- > 12 months.

The exception was for adverse event data, when we used the longest time period of follow‐up. 

We searched the COMET database for existing core outcome sets of relevance to Ménière's disease and vertigo, but were unable to find any published core outcome sets. We therefore conducted a survey of individuals with experience of (or an interest in) balance disorders to help identify the outcomes that should be prioritised. This online survey was conducted with the support of the Ménière's Society and the Migraine Trust, and included 324 participants, who provided information regarding priority outcomes. The review author team used the results of this survey to inform the choice of outcome measures in this review. 

We analysed the following outcomes in the review, but did not use them as a basis for including or excluding studies.

##### Primary outcomes

- Improvement in vertigo
  - Measured as a dichotomous outcome (improved/not improved), according to self‐report, or according to a change of a specified score (as described by the study authors) on a vertigo rating scale.
- Change in vertigo
  - Measured as a continuous outcome, to identify the extent of change in vertigo symptoms.
- Serious adverse events
  - Including any event that causes death, is life‐threatening, requires hospitalisation, results in disability or permanent damage, or in congenital abnormality. Measured as the number of participants who experience at least one serious adverse event during the follow‐up period.

Vertigo symptoms comprise a variety of different features, including frequency of episodes, duration of episodes and severity/intensity of the episodes. Where possible, we included data for the vertigo outcomes that encompassed all of these three aspects (frequency, duration and severity/intensity of symptoms). However, we anticipated that these data may not be available from all studies. We therefore extracted data on the frequency of vertigo episodes as an alternative measure for these outcomes. 

##### Secondary outcomes

- Disease‐specific health‐related quality of life
  - Measured with the Dizziness Handicap Inventory (DHI, Jacobsen 1990), a validated measurement scale in widespread use. If data from the DHI were unavailable we extracted data from alternative validated measurement scales, according to the order of preference described in the list below (based on the validity of the scales for this outcome):
    - DHI short form (Tesio 1999);
    - DHI screening tool (Jacobsen 1998);
    - Vertigo Handicap Questionnaire (Yardley 1992a);
    - Meniere's Disease Patient Oriented Symptoms Inventory (MD POSI, Murphy 1999);
    - University of California Los Angeles Dizziness Questionnaire (UCLADQ, Honrubia 1996);
    - AAO‐HNS Functional Level Scale (FLS, AAO‐HNS 1995).
- Hearing
  - Measured with pure tone audiometry and reported as the change in pure tone average (PTA), or (alternatively) by patient report, if data from PTA were not available.
- Tinnitus
  - Measured using any validated, patient‐reported questionnaire relating to the impact of tinnitus, for example the Tinnitus Handicap Inventory (THI, Newman 1996) or the Tinnitus Functional Index (TFI, Miekle 2012). 
- Other adverse effects
  - Measured as the number of participants who experienced at least one episode of the specified adverse events during the follow‐up period. Including the following specified adverse effects:
    - otitis media;
    - persistent perforated tympanic membrane;
    - ear discharge;
    - ear pain;
    - new onset, permanent and total hearing loss in the affected ear;
    - new onset of tinnitus in the affected ear.

### Search methods for identification of studies

The Cochrane ENT Information Specialist conducted systematic searches for randomised controlled trials and controlled clinical trials in October 2021 and 14 September 2022. There were no language, publication year or publication status restrictions. The date of the search was 14 September 2022.

#### Electronic searches

The Information Specialist searched:

- the Cochrane ENT Trials Register (search via the Cochrane Register of Studies to 14 September 2022);
- the Cochrane Central Register of Controlled Trials (CENTRAL) (search via the Cochrane Register of Studies to 14 September 2022);
- Ovid MEDLINE(R) Epub Ahead of Print, In‐Process & Other Non‐Indexed Citations, Ovid MEDLINE(R) Daily and Ovid MEDLINE(R) (1946 to 14 September 2022);
- Ovid Embase (1974 to 14 September 2022);
- Web of Knowledge, Web of Science (1945 to 14 September 2022);
- ClinicalTrials.gov, www.clinicaltrials.gov (to 14 September 2022);
- World Health Organization (WHO) International Clinical Trials Registry Platform (ICTRP), https://trialsearch.who.int/ (to 14 September 2022).

The Information Specialist modelled subject strategies for databases on the search strategy designed for CENTRAL. The strategy has been designed to identify all relevant studies for a suite of reviews on various interventions for Ménière's disease. Where appropriate, they were combined with subject strategy adaptations of the highly sensitive search strategy designed by Cochrane for identifying randomised controlled trials and controlled clinical trials (as described in the *Cochrane Handbook for Systematic Reviews of Interventions* Version 5.1.0, Box 6.4.b; Handbook 2011 [https://revman.cochrane.org/#/654321080213284837/dashboard/htmlView/1.1.23?revertEnabled=false&versionWithProductionChanges=false#REF‐Handbook‐2011]). Search strategies for major databases including CENTRAL are provided in Appendix 2 [https://revman.cochrane.org/#/654321080213284837/dashboard/htmlView/1.1.23?revertEnabled=false&versionWithProductionChanges=false#APP‐02].

#### Searching other resources

We scanned the reference lists of identified publications for additional trials and contacted trial authors where necessary. In addition, the Information Specialist searched Ovid MEDLINE to retrieve existing systematic reviews relevant to this systematic review, so that we could scan their reference lists for additional trials. The Information Specialist also ran a non‐systematic search of Google Scholar to identify trials not published in mainstream journals. 

We did not perform a separate search for adverse effects. We considered adverse effects described in included studies only.

### Data collection and analysis

#### Selection of studies

 The Cochrane ENT Information Specialist used the first two components of Cochrane's Screen4Me workflow to help assess the search results: 

1. Known assessments – a service that matches records in the search results to records that have already been screened in Cochrane Crowd and been labelled as 'a RCT' or as 'not a RCT'. 
2. The machine learning classifier (RCT model) (Wallace 2017), available in the Cochrane Register of Studies (CRS‐Web), which assigns a probability of being a true RCT (from 0 to 100) to each citation. Citations that were assigned a probability score below the cut‐point at a recall of 99% were assumed to be non‐RCTs. We manually dual screened the results for those that scored on or above the cut‐point. 

At least two review authors (BG, KG, KW) or co‐workers (AL and SC, listed in Acknowledgements) independently screened the remaining titles and abstracts using Covidence, to identify studies that may be relevant for the review. Any discrepancies were resolved by consensus, or by retrieving the full text of the study for further assessment. 

We obtained the full text for any study that was considered possibly relevant and two authors (BG, KG, KW) or a co‐worker (AL) again independently checked this to determine whether it met the inclusion criteria for the review. Any differences were resolved by discussion and consensus, or through recourse to a third author if necessary. 

We excluded any studies that were retrieved in full text but subsequently deemed to be inappropriate for the review (according to the inclusion/exclusion criteria), according to the main reason for exclusion. 

The unit of interest for the review is the study, therefore multiple papers or reports of a single study are grouped together under a single reference identification. The process for study selection is recorded in Figure 1. 

**1:**
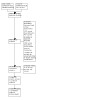
Flow chart of study retrieval and selection.

##### Screening eligible studies for trustworthiness

We assessed studies meeting our inclusion criteria for trustworthiness using a screening tool developed by Cochrane Pregnancy and Childbirth. This tool includes specified criteria to identify studies that are considered sufficiently trustworthy to be included in the review (see Appendix 2 and Figure 2). If studies were assessed as being potentially 'high‐risk', we attempted to contact the study authors to obtain further information or address any concerns. We planned to exclude studies from the main analyses of the review if there were persisting concerns over trustworthiness, or we were unable to contact the authors. 

**2:**
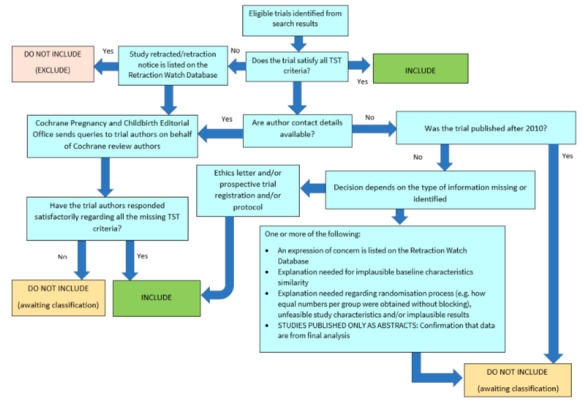
The Cochrane Pregnancy and Childbirth Trustworthiness Screening Tool

For this review, two included studies had some concerns when using the tool. We were unable to identify a prospective trial registration or obtain a copy of the trial protocol for either Gurkov 2012 or Russo 2017. This was also the case for Gates 2004, however this trial was published before 2010, and the trustworthiness screening tool does not require older studies to be pre‐registered.

We attempted to contact authors to clarify these issues, but we either received no reply, or the authors were unable to access the original trial data to clarify our queries. We had not anticipated this issue when drafting the protocol for our review, but it is likely to be a widespread issue for reviews that incorporate older studies, and has been a persistent problem through this suite of reviews on Ménière's disease.

There are several possible explanations for the large number of studies that had concerns when using the tool. One is that there are issues with the trustworthiness of the studies identified in this review, and the data included may not give reliable estimates of the true effect. Alternatively, the trustworthiness screening tool may be excessively sensitive, and flag studies that are trustworthy, but where information has not been fully reported. We note that this tool (and others used for the same purpose) has not yet been validated for use. 

We therefore took the decision to include the studies in the review, despite the potential concerns over trustworthiness. The uncertainty in the results is captured as part of our GRADE rating in the certainty of the evidence, using the domain 'study limitations'. 

#### Data extraction and management

Two review authors (BG, KW) independently extracted outcome data from each study using a standardised data collection form. Where a study had more than one publication, we retrieved all publications to ensure that we had a complete data set. We checked any discrepancies in the data extracted by the two authors against the original reports, and resolved differences through discussion and consensus. If required, we contacted the study authors for clarification.

We extracted data on the key characteristics of the studies, including the following information:

- study design, duration of the study, number of study centres and location, study setting and dates of the study;
- information on the participants, including the number randomised, those lost to follow‐up or withdrawn, the number analysed, the age of participants, gender, severity of the condition, diagnostic criteria used, inclusion and exclusion criteria for the individual studies;
- details of the intervention, comparator, and concomitant treatments or excluded medications;
- the outcomes specified and reported by the study authors, including the time points;
- funding for the study and any conflicts of interest for the study authors;
- information required to assess the risk of bias in the study, and to enable GRADE assessment of the evidence.

Once the extracted data were checked and any discrepancies resolved, a single author transferred the information to Review Manager 5 (RevMan 2020). 

The primary effect of interest for this review is the effect of treatment assignment (which reflects the outcomes of treatment for people who were assigned to the intervention) rather than a per protocol analysis (the outcomes of treatment only for those who completed the full course of treatment as planned). For the outcomes of interest in this review, we extracted the findings from the studies on an available case basis, i.e. all available data from all participants at each time point, based on the treatment to which they were randomised. This was irrespective of compliance, or whether participants had received the intervention as planned.

In addition to extracting pre‐specified information about study characteristics and aspects of methodology relevant to risk of bias, we extracted the following summary statistics for each study and outcome:

- For continuous data: the mean values, standard deviation and number of patients for each treatment group at the different time points for outcome measurement. Where change‐from‐baseline data were not available, we extracted the values for endpoint data instead. If values for the individual treatment groups were not reported, where possible we extracted summary statistics (e.g. mean difference) from the studies.
- For binary data: we extracted information on the number of participants experiencing an event, and the number of participants assessed at that time point. If values for the individual treatment groups were not reported, where possible we extracted summary statistics (e.g. risk ratio) from the studies.
- For ordinal scale data: if the data appeared to be normally distributed, or if the analysis performed by the investigators indicated that parametric tests are appropriate, then we treated the outcome measure as continuous data. Alternatively, if data were available, we converted these to binary data for analysis ‐ for example, for analysis of improvement in vertigo, when rated using the AAO‐HNS 1995 control of vertigo scale. 
- For time‐to‐event data: we did not identify any time‐to‐event data for the outcomes specified in the review. 

If necessary, we converted data found in the studies to a format appropriate for meta‐analysis, according to the methods described in the *Cochrane Handbook for Systematic Reviews of Interventions* (Handbook 2021). 

We pre‐specified time points of interest for the outcomes in this review. Where studies reported data at multiple time points, we took the longest available follow‐up point within each of the specific time frames. For example, if a study reported an outcome at 12 weeks and 20 weeks of follow‐up then we included the 20‐week data for the time period 3 to 6 months (12 to 24 weeks).

#### Assessment of risk of bias in included studies

Two authors (BG, KW) undertook assessment of the risk of bias of the included studies independently, with the following taken into consideration, as guided by the *Cochrane Handbook for Systematic Reviews of Interventions* (Handbook 2011):

- sequence generation;
- allocation concealment;
- blinding;
- incomplete outcome data;
- selective outcome reporting; and
- other sources of bias.

We used the Cochrane risk of bias tool (Handbook 2011), which involves describing each of these domains as reported in the study and then assigning a judgement about the adequacy of each entry: 'low', 'high' or 'unclear' risk of bias.

#### Measures of treatment effect

We summarised the effects of the majority of dichotomous outcomes (e.g. serious adverse effects) as risk ratios (RR) with 95% confidence intervals (CIs). We have also expressed the results as absolute numbers based on the pooled results and compared to the assumed risk in the summary of findings table (Table 1) and full GRADE profile (Table 2). 

**1:** GRADE profile: positive pressure therapy compared to placebo

**Certainty assessment**							**Number of participants**		**Effect**		**Certainty**	**Comment**
**№ of studies**	**Study design**	**Risk of bias**	**Inconsistency**	**Indirectness**	**Imprecision**	**Other considerations**	**Positive pressure therapy**	**Placebo**	**Relative** **(95% CI)**	**Absolute** **(95% CI)**		
**Improvement in vertigo frequency (follow‐up: range 3 months to 6 months)**												
1	Randomised trials	Serious^a^	Not serious	Not serious	Serious^b^	None	27/41 (65.9%)	20/36 (55.6%)	**RR 1.19** (0.82 to 1.71)	**106 more per 1000** (from 100 fewer to 394 more)	⨁⨁◯◯ Low	Positive pressure therapy may slightly increase the proportion of people in whom the frequency of vertigo improves at 3 to < 6 months.
**Change in vertigo (global score) (follow‐up: range 3 months to < 6 months; assessed with: cumulative vertigo score in a 4‐week period; scale from: 0 to 112, lower = better)**												
1	Randomised trials	Serious^c^	Not serious	Serious^d^	Serious^b^	None	37	31	‐	MD **5.31 points lower** (11.67 lower to 1.05 higher)	⨁◯◯◯ Very low	The evidence is very uncertain regarding the effect of positive pressure therapy on global scores of vertigo at 3 to < 6 months.
**Change in frequency of vertigo (follow‐up: range 3 months to <6 months; assessed with: Number of days with definitive vertigo episodes)**												
3	Randomised trials	Very serious^e^	Not serious	Not serious	Serious^b^	None	107	95	‐	MD **0.84 days per month lower** (2.12 lower to 0.45 higher)	⨁◯◯◯ Very low	The evidence is very uncertain regarding the effect of positive pressure therapy on the change in frequency of vertigo at 3 to < 6 months.
**Disease‐specific health‐related quality of life (3 to < 6 months) (follow‐up: range 3 months to < 6 months; assessed with: AAO‐HNS 1995 FLS; scale from: 1 to 6)**												
1	Randomised trials	Serious^a^	Not serious	Not serious	Serious^b^	None	41	36	‐	MD **0 points ** (0.1 lower to 0.1 higher)	⨁⨁◯◯ Low	Positive pressure therapy may make little or no difference to disease‐specific health‐related quality of life at 3 to 6 months.
**Hearing (3 to < 6 months)**												
2	Randomised trials	Very serious^e^	Serious^f^	Not serious	Serious^b^	None	65	58	‐	MD **2.49 dB HL higher** (6.17 lower to 11.14 higher)	⨁◯◯◯ Very low	The evidence is very uncertain regarding the effect of positive pressure therapy on hearing at 3 to < 6 months.

**AAO‐HNS:** American Academy of Otolaryngology ‐ Head and Neck Surgery; **CI:** confidence interval; **dB HL:** decibel hearing level; **FLS:** Functional Level Scale; **MD:** mean difference; **RR:** risk ratio ^a^Serious risk of attrition bias and selective reporting. ^b^Sample size fails to meet optimal information size, taken as < 400 participants for a continuous outcome, or < 300 events for a dichotomous outcome, as a rule of thumb. ^c^Serious risk of attrition bias. ^d^Risk of bias due to use of an unvalidated scale for this outcome. ^e^Serious risk of attrition bias. Some concerns over selective reporting and other bias. ^f^I^2^ = 45%. Effect favours control in one study and shows equivalence between the interventions in the other study.

For continuous outcomes, we expressed treatment effects as a mean difference (MD) with standard deviation (SD). We did not need to use the standardised mean difference to pool any data. 

#### Unit of analysis issues

Ménière's disease is unlikely to be a stable condition, and interventions may not have a temporary effect. If cross‐over trials had been identified then we planned to use the data from the first phase of the study only. If cluster‐randomised trials were identified then we would have ensured that analysis methods were used to account for clustering in the data (Handbook 2021). Similarly, if we identified studies with three or more arms, we planned to ensure these were included to avoid double‐counting of any participants. However, all the trials identified had only two arms, and we did not identify any cross‐over or cluster‐randomised trials for this review. 

#### Dealing with missing data

We planned to contact study authors via email whenever the outcome of interest was not reported, if the methods of the study suggest that the outcome had been measured. We did the same if not all data required for meta‐analysis were reported (for example, standard deviations), unless we were able to calculate them from other data reported by the study authors. 

#### Assessment of heterogeneity

We assessed clinical heterogeneity by examining the included studies for potential differences between them in the types of participants recruited, interventions or controls used and the outcomes measured. This is highlighted in the Included studies section, below.

We used the I^2^ statistic to quantify inconsistency among the trials in each meta‐analysis. We also considered the P value from the Chi^2^ test. However, few meta‐analyses were conducted in the course of this review, and we did not identify any serious inconsistency. 

#### Assessment of reporting biases

We assessed reporting bias as within‐study outcome reporting bias and between‐study publication bias.

##### Outcome reporting bias (within‐study reporting bias)

We assessed within‐study reporting bias by comparing the outcomes reported in the published report against the study protocol or trial registry, whenever this could be obtained. If the protocol or trial registry entry was not available, we compared the outcomes reported to those listed in the methods section. If results are mentioned but not reported adequately in a way that allows analysis (e.g. the report only mentions whether the results were statistically significant or not), bias in a meta‐analysis is likely to occur. We then sought further information from the study authors. If no further information was found, we noted this as being a 'high' risk of bias with the risk of bias tool. If there was insufficient information to judge the risk of bias we noted this as an 'unclear' risk of bias (Handbook 2011). 

##### Publication bias (between‐study reporting bias)

We did not have sufficient studies to create funnel plots for any analysis. Any studies identified through trial registries and other sources (Searching other resources) that remain unpublished are noted in the Ongoing studies section. 

#### Data synthesis

##### Meta‐analysis of numerical data

Where possible and appropriate (if participants, interventions, comparisons and outcomes were sufficiently similar in the trials identified) we conducted a quantitative synthesis of results. We conducted all meta‐analyses using RevMan 2020. We anticipated that the underlying effect of the intervention may vary between studies, as there are likely to be differences between participants, settings and the interventions used for each study. Therefore, we used a random‐effects method for meta‐analysis. We explored whether the use of a fixed‐effect model substantially altered the effect estimates (see Sensitivity analysis). 

For dichotomous data, we analysed treatment differences as a risk ratio (RR) calculated using the Mantel‐Haenszel methods. For continuous outcomes, we pooled mean follow‐up values with change‐from‐baseline data and reported this as a mean difference.

Improvement in vertigo symptoms may be assessed using a variety of methods, which consider different aspects of vertigo. These include:

- frequency of vertigo episodes;
- duration of vertigo episodes;
- severity/intensity of vertigo episodes;
- a composite measure of all of these aspects:
  - for example, assessed with a global score ‐ such as "how troublesome are your vertigo symptoms?", rated on an ordinal scale.

For the outcomes "improvement in vertigo" and "change in vertigo", we prioritised outcome measures that used a composite score ‐ encompassing aspects of vertigo frequency, duration and severity/intensity. Examples of this may include a global rating scale of vertigo impact (rated from 0 to 10, where 0 is defined as no symptoms, and 10 is defined as the most troublesome symptoms) or the vertigo/balance subscale of the Vertigo Symptom Scale (Yardley 1992b), or Vertigo Symptom Scale Short Form (Yardley 1998). As data from composite scores were not available from the majority of studies, we also included data on the frequency of vertigo episodes as an alternative measure.

##### Synthesis using other methods

If we were unable to pool numerical data in a meta‐analysis for one or more outcomes we planned to provide a synthesis of the results using alternative methods, following the guidance in chapter 12 of the Handbook 2021. However, this was not necessary, as results were typically provided by a single study. 

#### Subgroup analysis and investigation of heterogeneity

If statistical heterogeneity was identified for any comparison, we planned to assess this considering the following subgroups:

- Different comparators.
- Different pressures/duration/frequency of administration.
- Use of concomitant treatment.
- Diagnosis of Ménière's disease.

However, due to the paucity of data available, and the few meta‐analyses included in this review, we did not carry out any subgroup analysis. 

#### Sensitivity analysis

We planned to carry out a number of sensitivity analyses for the primary outcomes in this review. However, the paucity of data and the lack of meta‐analyses has meant that this was not possible. 

If few studies are identified for meta‐analysis, the random‐effects model may provide an inaccurate measure of the between‐studies variance. Therefore, we explored the impact of using a fixed‐effect model using a sensitivity analysis ‐ the results are very similar (Table 3).

**2:** Sensitivity analysis

**Analysis**	**Sensitivity analysis**	**Result**
Analysis 1.3 Change in vertigo frequency at 3 to < 6 months	Fixed‐effect model	MD ‐0.84 (95% CI ‐2.12 to 0.45)
Analysis 1.5 Change in hearing at 3 to < 6 months	Fixed‐effect model	MD 0.94 (95% CI ‐3.93 to 5.81)

CI: confidence interval; MD: mean difference

If there was uncertainty over the diagnostic criteria used for participants in the studies (for example, if it is not clear whether participants were diagnosed using criteria that are analogous to the AAO‐HNS criteria) then we also planned to explore this by including/excluding those studies from the analysis. However, all the included studies reported inclusion of participants with definite Ménière's disease, according to the AAO‐HNS 1995 guidelines. 

We used the Cochrane Pregnancy and Childbirth Screening Tool to identify any studies with concerns over the data available. We had intended that any studies identified by the tool would be excluded from the main analyses in the review, but that we would explore the impact of including the data from these studies through a sensitivity analysis. However, as noted above, we had some concerns over the use of this tool, and few studies were included in the review, therefore this sensitivity analysis was not conducted. 

#### Summary of findings and assessment of the certainty of the evidence

Two independent authors (KG, KW) used the GRADE approach to rate the overall certainty of evidence using GRADEpro GDT (https://gradepro.org/) and the guidance in chapter 14 of the *Cochrane Handbook for Systematic Reviews of Interventions* (Handbook 2021). Disagreements were resolved through discussion and consensus. The certainty of evidence reflects the extent to which we are confident that an estimate of effect is correct, and we have applied this in the interpretation of results. There are four possible ratings: high, moderate, low and very low. A rating of high certainty of evidence implies that we are confident in our estimate of effect and that further research is very unlikely to change our confidence in the estimate of effect. A rating of very low certainty implies that any estimate of effect obtained is very uncertain.

The GRADE approach rates evidence from RCTs that do not have serious limitations as high certainty. However, several factors can lead to the downgrading of the evidence to moderate, low or very low. The degree of downgrading is determined by the seriousness of these factors:

- Study limitations (risk of bias):
  - This was assessed using the rating from the Cochrane risk of bias tool for the study or studies included in the analysis. We rated down either one or two levels, depending on the number of domains that had been rated at high or unclear risk of bias. 
- Inconsistency:
  - This was assessed using the I^2^ statistic and the P value for heterogeneity for all meta‐analyses, as well as by visual inspection of the forest plot. For results based on a single study we rated this domain as no serious inconsistency.
- Indirectness of evidence:
  - We took into account whether there were concerns over the population included in these study or studies for each outcome, as well as whether additional treatments were offered that may impact on the efficacy of the intervention under consideration. 
- Imprecision:
  - We took into account the sample size and the width of the confidence interval for each outcome. If the sample size did not meet the optimal information size (i.e. < 400 people for continuous outcomes or < 300 events for dichotomous outcomes), or the confidence interval crossed the small effect threshold, we rated down one level. If the sample size did not meet the optimal information size and the confidence interval includes both potential harm and potential benefit we rated down twice. We also rated down twice for very tiny studies (e.g. 10 to 15 participants in each arm), regardless of the estimated confidence interval.
- Publication bias:
  - We considered whether there were likely to be unpublished studies that may impact on our confidence in the results obtained. 

We used a minimally contextualised approach, and rated the certainty in the interventions having an important effect (Zeng 2021). Where possible, we used agreed minimally important differences (MIDs) for continuous outcomes as the threshold for an important difference. Where no MID was identified, we provide an assumed MID based on agreement between the authors. For dichotomous outcomes, we looked at the absolute effects when rating imprecision, but also took into consideration the GRADE default approach (rating down when a RR crosses 1.25 or 0.80). We have justified all decisions to downgrade the certainty of the evidence using footnotes, and added comments to aid the interpretation of the findings, where necessary. 

We provide a summary of findings table for the following comparison:

- positive pressure therapy versus placebo/no treatment.

We have included all primary outcomes in the summary of findings table. We have also included a full GRADE profile for all results and comparisons (see Table 2). 

## Results

### Description of studies

#### Results of the search

The searches in October 2021 and September 2022 retrieved a total of 4434 records. This reduced to 3408 after the removal of duplicates. The Cochrane ENT Information Specialist sent all 3408 records to the Screen4Me workflow. The Screen4Me workflow identified 122 records as having previously been assessed: 83 had been rejected as not RCTs and 39 had been assessed as possible RCTs. The RCT classifier rejected an additional 1427 records as not RCTs (with 99% sensitivity). We did not send any records to the Cochrane Crowd for assessment. Following this process, the Screen4Me workflow had rejected 1510  records and identified 1898 possible RCTs for title and abstract screening. 

**Table:** 

** **	**Possible RCTs**	**Rejected**
Known assessments	39	83
RCT classifier	1859	1427
Total	1898	1510

We identified 89 additional duplicates. We screened the titles and abstracts of the remaining 1809 records. We discarded 1790 records and assessed 19 full‐text records. We were unable to locate the full text of four conference abstracts (four records), and these are listed in Studies awaiting classification.

We excluded 13 records (linked to 12 studies) with reasons recorded in the review (see Excluded studies). We included three completed studies (six records) where results were available.

A flow chart of study retrieval and selection is provided in Figure 1.

#### Included studies

We included three RCTs (Gates 2004; Gurkov 2012; Russo 2017). Details of individual studies can be found in the Characteristics of included studies.

##### Study design

All included studies were parallel‐group, randomised, controlled trials. The duration of follow‐up ranged from 12 weeks (Russo 2017) to four months (Gates 2004; Gurkov 2012). One trial was conducted in the USA (Gates 2004), one in Germany (Gurkov 2012) and one in France (Russo 2017).

##### Participants

All the included studies recruited adult participants. For all studies, participants were required to have diagnosis of definite Ménière's disease, according to the AAO‐HNS 1995 criteria.

###### Features of Ménière's disease

All three studies specifically stated that participants with only unilateral disease were included, and required participants to have a minimum frequency of two vertigo episodes per month before enrolment into the study. The average duration of Ménière's symptoms varied:

- Most participants in Russo 2017 had symptoms for less than one year.
- Gurkov 2012 included participants who had symptoms for approximately one to two years.
- The median duration of symptoms in Gates 2004 was 4.5 years. 

###### Background interventions

The background treatments varied across the studies. Most participants in Gates 2004 were receiving diuretics as maintenance treatment, and were allowed to continue this treatment for the duration of the trial. Conversely, participants in Gurkov 2012 were all receiving betahistine (at different doses), and also maintained this therapy for the duration of the trial. 

The study Russo 2017 reports the use of a 'wash‐out' period of eight weeks at the start of the trial. We assume this means that any maintenance treatments were discontinued. 

##### Interventions and comparisons

###### Positive pressure therapy compared to no treatment/placebo

All the included studies used the Meniett device (Medtronic Xomed, Jacksonville Florida) to provide positive pressure treatment. The Meniett device is a small machine that generates a sequence of low‐pressure waves. These are transported to the ear through tubing, connected to an earpiece. People undergoing treatment with this device need a ventilation tube inserted in the ear first, to allow the pressure wave to conduct through to the inner ear. This was compared to the use of an inactive device in the control group. Two studies reported that the inactive device produced no pressure increase (Gates 2004; Russo 2017), but one study used a very slight pressure increase in the control group (to 2 cm H_2_O; Gurkov 2012). Participants in both groups (active and inactive device) had a ventilation tube inserted at the start of the trial.

Gates 2004 and Gurkov 2012 used an identical regime for the Meniett device. It was to be used three times daily, for five minutes each time. A 0.6‐second pressure pulse was used at 6 Hz, within the range of 0 cm to 20 cm H_2_O. For each five‐minute period, the pressure pulses were delivered for one minute, followed by a 40‐second pause. This cycle was repeated three times. 

Russo 2017 used a different technique. The device was also used three times daily, but for 15 minutes each time. They state that pressure pulses were at a frequency of 6 Hz, with a maximum pressure of 12 cm H_2_O. 

It should be noted that Russo 2017 reported outcomes when participants had stopped using the Meniett device. Study participants used the device for six weeks, then stopped for six weeks, and outcomes were reported at 12 weeks of follow‐up. This was in contrast to the other studies, where outcomes were reported whilst participants were receiving active treatment. 

##### Outcomes

###### 1. Improvement in vertigo

For this outcome we included dichotomous data ‐ assessed as the proportion of participants whose vertigo had 'improved' or 'not improved'. 

####### 1.1. Global score

No studies reported the improvement of vertigo using a global score that considered the frequency, duration and intensity of vertigo attacks. 

####### 1.2. Frequency

One study assessed improvement in vertigo frequency. No minimum change in frequency was required for this study. Any participant in whom the frequency of vertigo episodes was lower at final follow‐up than at baseline was considered to have improved.  

###### 2. Change in vertigo

This outcome included data on the change in vertigo using a continuous numerical scale. 

####### 2.1. Global score

A single study assessed the change in vertigo using a global score (Gurkov 2012). They used a score which was previously developed by Gates 2004. Participants were asked to score vertigo‐free days as 0, days with a mild attack as 1, days with moderately severe attacks (lasting more than 20 minutes) as 2, days with severe attacks lasting longer than one hour (and accompanied by nausea and vomiting) as 3, and the worst attack ever experienced as 4. The total symptom score in a given period of time therefore incorporates aspects of the duration, frequency and severity of vertigo. We have been unable to establish whether this score is a validated method to measure vertigo severity and impact. 

####### 2.2. Frequency

All included studies assessed the change in the frequency of vertigo over a period of time. The time period varied across the studies, but we were able to convert these to the same scale to enable meta‐analysis of the results. Gates 2004 reported on the proportion of days with a "definitive vertigo episode" (lasting greater than 20 minutes) per month. We converted this to an absolute number of episodes in a 28‐day period. Gurkov 2012 reported on the change from baseline in the number of days of vertigo experienced per month. For the purposes of analysis we assumed that this was the number of episodes in a 28‐day period, although we note that this may be a slight under‐estimate for both groups. Finally, Russo 2017 reported on the number of vertigo episodes in a 21‐day period; we re‐scaled these data to a 28‐day period. 

###### 3. Serious adverse events

None of the studies provided any details on how or whether serious adverse events were monitored and reported. 

###### 4. Disease‐specific health‐related quality of life

One study considered this outcome, and reported the Functional Level Scale (FLS) score from the AAO‐HNS 1995 guidelines. This ranges from 1 to 6, with higher scores representing worse quality of life. 

###### 5. Hearing

Pure tone audiometry (PTA) was used to assess hearing status in two studies. Both used a pure tone average from three frequencies (0.25 kHz, 0.5 kHz and 1 kHz). 

###### 6. Tinnitus 

This outcome was not reported by any of the included studies. 

###### 7. Other adverse effects

None of the studies provided any details on how adverse effects were monitored and reported. It is unclear whether the specific adverse effects that were prioritised in this review would have been assessed and reported as part of the studies or not. 

#### Excluded studies

After assessing the full text, we excluded 12 articles from this review. The main reason for exclusion for each article is listed below.

Six studies were not randomised controlled trials (Barbara 2001; UMIN000002356; UMIN000002357; UMIN000005544; UMIN000007659; UMIN000017710).

Three studies were narrative review articles on Ménière's disease and/or positive pressure therapy, and did not report any primary data (Conde 1965; Odkvist 2004; Richards 1971).

Finally, three randomised controlled trials were identified, but they had an insufficient duration of follow‐up for inclusion in this review ‐ we required all studies to have followed participants up for a minimum of three months. These were:

- Densert 1997, which reported outcomes immediately after the use of a positive pressure device (same day); 
- Odkvist 2000, which reported outcomes at two weeks of follow‐up; and
- Thomsen 2005, which reported outcomes at a maximum of eight weeks follow‐up. 

### Risk of bias in included studies

See Figure 3 for the risk of bias graph (our judgements about each risk of bias item presented as percentages across all included studies) and Figure 4 for the risk of bias summary (our judgements about each risk of bias item for each included study). All the studies included had some concerns regarding the risk of bias, with at least two domains being rated at high risk of bias. 

**3:**
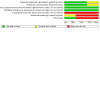
Risk of bias graph (our judgements about each risk of bias item presented as percentages across all included studies).

**4:**
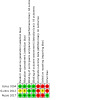
Risk of bias summary (our judgements about each risk of bias item for each included study).

#### Allocation

Two included studies reported the methods used to randomised participants and conceal their allocation to groups sufficiently clearly to be rated at low risk of bias for this domain (Gates 2004; Russo 2017). The third study did not provide any information on the randomisation method, or procedures put in place to conceal allocation, therefore we judged it at unclear risk of bias for these domains (Gurkov 2012). 

#### Blinding

All three studies described the use of a placebo device, and indicated that participants and study personnel were blinded to the group allocation. It should be noted that the placebo device used did not generate any pressure increase for two studies (Gates 2004; Russo 2017), and only generated a very small pressure increase for the third study (Gurkov 2012). We were uncertain whether participants using the devices would therefore be aware of the pressure sensation (or lack of this sensation) and may inadvertently become aware of their group allocation. One of the included studies provided some information on this, and stated that "patients were unable to detect whether they were using the active or the placebo device" (Gurkov 2012). On this basis, we have rated performance and detection bias as low risk for all the studies. However, we do note the differential dropout between the two groups (see below), which may be ‐ in part ‐ because of awareness of group allocation.

#### Incomplete outcome data

Attrition bias was a concern for all the included studies. In Gates 2004, participants were able to self‐declare "treatment failure" at the two‐month follow‐up. They were then excluded from analysis at four months. These exclusions occurred at a greater rate in the placebo group (four participants) than the active treatment group (one participant). Dropout in Gurkov 2012 and Russo 2017 was also substantially higher in the placebo group as compared to the active intervention group.

#### Selective reporting

We rated two studies at high risk of bias for selective reporting. Russo 2017 indicated that data on the severity of vertigo episodes were collected, but this is not reported in the publication. A global score of vertigo severity was also used by Gates 2004, but the data are reported in a way that precludes meta‐analysis (the sum of vertigo scores for the entire group is reported, rather than mean scores for individuals). 

We were also unable to identify a prospective trial registration or protocol for Gurkov 2012 (registration with clinicaltrials.gov was retrospective), so we rated this at unclear risk of selective reporting bias.

#### Other potential sources of bias

An unvalidated scoring system was used to assess vertigo in two studies, therefore we considered this to be a potential additional source of bias (Gates 2004; Gurkov 2012). 

### Effects of interventions

See: Table 1

#### 1. Positive pressure compared to no treatment/placebo

##### 1.1. Improvement in vertigo

###### 1.1.1. Improvement in global score

No studies measured global improvement in vertigo ‐ taking account of the frequency, severity or intensity and duration of symptoms. 

###### 1.1.2. Improvement in frequency

A single study assessed improvement in vertigo frequency. Participants were considered to have improved if the frequency of vertigo episodes at the end of treatment (three months) was lower than that recorded at baseline. 

####### 1.1.2.1. 3 to < 6 months

More participants reported improvement in the frequency of vertigo episodes with positive pressure therapy than with placebo, however the confidence intervals were very wide and include the possibility of no effect. The risk ratio (RR) for improvement was 1.19 (absolute effects 65.9% positive pressure group, 55.6% control group, RR 1.19, 95% confidence interval (CI) 0.82 to 1.71; 1 study; 77 participants; low‐certainty evidence; Analysis 1.1). 

####### 1.1.2.2. 6 to ≤ 12 months and > 12 months

No studies reported this outcome during these time periods. 

##### 1.2. Change in vertigo

###### 1.2.1. Change in global score

A single study reported on the change in vertigo using a global score, which included the frequency of episodes, the severity or intensity of symptoms and the duration of episodes (Gurkov 2012). For this study, scores were assessed over a four‐week period, leading to a potential total score of 0 to 112 (higher scores indicating worse symptoms).

####### 1.2.1.1. 3 to < 6 months

Global scores of vertigo were lower for those who received positive pressure than those receiving the control intervention, with a mean difference of ‐5.31 points (95% CI ‐11.67 to 1.05; range of scores 0 to 112, higher scores = worse symptoms; 1 study; 78 participants; very low‐certainty evidence; Analysis 1.2). 

####### 1.2.1.2. 6 to ≤ 12 months and > 12 months

No studies reported this outcome during these time periods. 

###### 1.2.2. Change in frequency

All three studies provided some information on the change in frequency of vertigo episodes. We were able to convert the data reported to an equivalent scale for each publication, and therefore report a pooled estimate of effect. 

####### 1.2.2.1. 3 to < 6 months

The frequency of vertigo episodes was slightly reduced in those who received positive pressure therapy, with a mean difference of ‐0.84 episodes per 28 days (95% CI ‐2.12 to 0.45; 3 studies; 202 participants; I^2^ = 0%; very low‐certainty evidence). 

####### 1.2.2.2. 6 to ≤ 12 months and > 12 months

No studies reported this outcome during these time periods. 

##### 1.3. Serious adverse events

Two of the included studies state that no adverse events occurred (Gates 2004; Gurkov 2012). However, it is not clear whether serious adverse events were specifically assessed and reported as part of the study. Adverse events were not commented on in the article by Russo 2017. 

##### 1.4. Disease‐specific health‐related quality of life

A single study assessed this outcome, using the Functional Level Scale (FLS) from the AAO‐HNS 1995 guidelines. The score ranges from 1 to 6, with higher scores representing worse quality of life. 

###### 1.4.1. 3 to < 6 months

There was no difference in the FLS score between those who received and did not receive positive pressure therapy, with a mean difference of 0 points (95% CI ‐0.1 to 0.1; 1 study; 77 participants; low‐certainty evidence; Analysis 1.4). 

###### 1.4.2. 6 to ≤ 12 months and > 12 months

No studies reported this outcome during these time periods. 

##### 1.5. Change in hearing

Two studies assessed hearing using pure tone audiometry.

###### 1.5.1. 3 to < 6 months

The hearing threshold was very slightly higher (worse) in those who had received positive pressure therapy, compared to those who had received placebo, with a difference of 2.49 dB HL (95% CI ‐6.17 to 11.14; 2 studies; 123 participants; I^2^ = 45%; very low‐certainty evidence; Analysis 1.5). There was some heterogeneity in this analysis, with one study showing a trivial difference between the two groups, and the other showing worse hearing for those receiving positive pressure therapy. 

###### 1.5.2. 6 to ≤ 12 months and > 12 months

No studies reported this outcome during these time periods.

##### 1.6. Change in tinnitus

No studies reported on this outcome. 

##### 1.7. Other adverse effects

As described above, two studies stated that no adverse effects occurred, but it is not clear whether the specific adverse effects of relevance to this review were assessed (or would have been reported). 

## Discussion

### Summary of main results

Positive pressure therapy may slightly increase the proportion of people who experience an improvement in the frequency of vertigo at 3 to < 6 months. However, the evidence on the actual change in vertigo frequency, and global scores of vertigo, was very uncertain. 

Positive pressure therapy may make little or no difference to disease‐related quality of life at 3 to 6 months. The evidence is very uncertain regarding the effect of positive pressure therapy on hearing at 3 to < 6 months, and we did not identify any evidence regarding the effect of treatment on tinnitus. 

We did not identify any evidence on serious adverse events, or other (less serious) adverse effects related to positive pressure therapy, so cannot comment on potential harms of this treatment. 

### Overall completeness and applicability of evidence

This review was conducted as part of a suite considering different interventions for Ménière's disease. A number of issues were identified as affecting the completeness and applicability of the evidence in all the reviews in this suite. These have been described in the companion review on systemic pharmacological interventions for Ménière's disease (Webster 2021a) and are replicated here, as they relate to this review:

- There is a paucity of evidence about all of these interventions, despite some of them being in common use for Ménière’s disease. All the evidence we found was of very low or low certainty, showing that we are unsure of the effects of the interventions, and future research may change the effect estimates a great deal.
- We were unable to carry out many meta‐analyses. There were often differences in the actual outcomes assessed in the study, therefore we were unable to pool the data to achieve a more precise estimate of any effect. Study authors also often used different ways of measuring the same outcome, which prevented data from being combined. For example, vertigo was assessed with either a global score, or a frequency score, which could not be combined.
- Certain outcomes were only assessed by some included studies. Some studies did not assess the impact of the disease on quality of life or tinnitus at all. Potential adverse effects of the interventions were also poorly reported or simply not assessed. The use of a positive pressure device requires the insertion of a ventilation tube. When used in people with chronic middle ear disease, this procedure has the potential to cause adverse effects (such as infection, ear discharge or persistent tympanic membrane perforation). It is unclear whether the same risks apply for people with Ménière's disease, but these should be assessed and reported. 
- We noted that unvalidated rating scales were commonly used in the studies included, particularly when looking at the global impact of treatments for vertigo. When such scales are used, it is difficult to know if they are accurately assessing the outcome, and also what size of change on this scale represents a meaningful difference in the outcome (the minimally important difference). 
- Finally, studies often failed to report clearly what treatments participants received before joining the trial, what maintenance treatment they continued on during the trial, and whether they received any additional treatments over the course of the trial. The impact of these additional treatments may be considerable, particularly for those studies with longer‐term follow‐up. Without knowing the background details of study participants (for example, the duration of their Ménière's disease, or what treatments they have tried in the past) it is difficult to identify the groups of people who may benefit from these treatments. 

### Quality of the evidence

We used the GRADE approach to assess the certainty of the evidence in this review. The evidence identified was all low‐ or very low‐certainty, meaning that we are uncertain about the actual effect of these interventions for all of our outcomes. The main issues which affected the certainty of the evidence were the domains of study limitations and imprecision. The different domains addressed by GRADE are considered in more detail below.

#### Study limitations/risk of bias

All the studies included in this review had at least some concerns regarding the potential for bias in the study design, conduct or reporting. Attrition bias and selective reporting were particular concerns for this review. We considered that blinding was adequate for the three studies included in the review ‐ as all studies reported the use of a sham device in the control group. However, it is possible that participants would be aware of the different pressure sensations generated by an active or a sham device, which would increase the potential for bias in these study results. 

#### Inconsistency

Few meta‐analyses were conducted in the course of this review, therefore inconsistency did not usually impact on the certainty of the evidence. 

#### Indirectness

This was not a major concern for most of the outcomes. We rated down for indirectness if there was significant concern over the methods used to measure an outcome (for example, use of an unvalidated scoring system for vertigo, as in Gurkov 2012).

#### Imprecision

All of the included studies are very small and, as discussed above, we were unable to carry out many meta‐analyses. Therefore, the total sample size for each of our outcomes of interest was small, and reduced the certainty of the evidence. For some outcomes the resulting confidence intervals for the effect size were also extremely wide ‐ meaning that there was uncertainty over whether the intervention was beneficial or harmful. This further impacted on the certainty of the evidence. 

For each analysis result, the width of the confidence interval is compared to the threshold for an important difference (details of how these thresholds were selected are described in the Methods section). If the confidence interval crosses this threshold ‐ and includes both the potential for an important benefit and the potential for a trivial effect, then the certainty of the evidence would be reduced by one level. If the confidence interval includes the possibility of *both* an important benefit and an important harm then the certainty would be reduced further.  Therefore, it is important to agree on thresholds for this rating, i.e. where is the threshold, or cut‐point, between a trivial difference and a small, but important benefit or harm for each outcome? This question is difficult to answer, and requires input from people with balance disorders. As part of this review process, one of the author team (KW) joined some discussion groups for people with balance disorders, to try and obtain their views on quantifying an important and meaningful difference in treatment outcomes. However, the main theme that emerged from these discussions was that people were unable to give a specific threshold for each outcome. Instead, individuals tended to weigh up a variety of different factors when determining this threshold. The invasiveness and burden of taking the treatment would be taken into account, as well as potential side effects and the severity of their symptoms at that time. The GRADE working group would likely refer to this as a "fully contextualised approach", accounting for all aspects of the specific intervention in order to set thresholds for benefit (Zeng 2021). For this review we adopted a "minimally contextualised approach" and rated imprecision for each outcome according to specific, defined thresholds (as described in Methods). However, if the thresholds used are inappropriate then this may affect the certainty of the evidence (by a maximum of one level). 

#### Other considerations

We did not rate down the certainty of the evidence for other reasons. Publication bias is usually assessed as part of this domain. Although we are aware that this is an issue with many systematic reviews, we did not find strong indications of publication bias with this review. We were unable to access the full text for a number of conference abstracts (see Characteristics of studies awaiting classification), therefore it is possible that this presents a risk of bias in the results. However, we did not include this as part of our GRADE assessment. 

### Potential biases in the review process

We planned to use the Cochrane Pregnancy and Childbirth Trustworthiness Tool to assess the included studies. We had intended to exclude any study where there were concerns (as identified with this tool) from the main analyses. However, as described above, we were unable to determine whether most of the included studies would pass the screening tool, either due to a lack of reporting in the original articles, or because we were unable to contact the authors to resolve any issues. If these studies were subsequently found to have genuine concerns over research integrity then this would further undermine our confidence in the findings of the review. However, as the evidence for these interventions is all low‐ or very low‐certainty, we considered that this would not greatly impact the findings of the review. 

As stated in our protocol, we only included studies if participants had been followed up for a minimum of three months (Webster 2021b). We did identify a small number of RCTs in which participants had been followed up for a shorter period of time, and these were consequently excluded from the review (Densert 1997; Odkvist 2000; Thomsen 2005). It is possible that inclusion of these data may have impacted on the review findings. However, with a chronic condition such as Ménière's disease, we felt that it was not appropriate to assess outcomes after such short periods of follow‐up.

### Agreements and disagreements with other studies or reviews

Many other published reviews in this area give similar conclusions to this review ‐ that the evidence for efficacy of positive pressure treatment is limited and uncertain. This includes a previous Cochrane Review on the same topic (van Sonsbeek 2015) and a number of other reviews (Devantier 2019; Holmberg 2019; Syed 2015). These reviews also include data from earlier time points, as they included studies with any duration of follow‐up.

We identified three reviews that included prospective cohort studies (using a before‐and‐after design) in their analyses (Ahsan 2015; Wang 2019; Zhang 2016). These all identified an improvement in vertigo symptoms with positive pressure therapy, although one stated that the efficacy may be limited (Wang 2019). Given the fluctuating nature of symptoms of Ménière's disease, we consider that it is difficult to draw conclusions of efficacy using studies with a before‐and‐after design. The inclusion of different study designs may have led to the different results between these previous reviews and the current review.

## Authors' conclusions

**Implications for practice**

At present, there is scarce information on the efficacy, and potential harms, of positive pressure therapy for Ménière's disease. Few randomised controlled trials (RCTs) have been conducted in this area, and the efficacy of treatment has only been assessed in a relatively small number of people. 

**Implications for research**

This review was conducted as part of a suite regarding different interventions for Ménière's disease. Many of the conclusions below are relevant to all of these reviews and are replicated across the suite.

The lack of high‐certainty, RCT evidence for positive pressure therapy suggests that well‐conducted studies with larger numbers of participants are required to appropriately assess the efficacy (and potential harms) of this intervention. However, there also needs to be more clarity on which outcomes studies should assess, when and how to assess them. Vertigo is a notoriously difficult symptom to assess, and there is great variety in the methods used to record and report this symptom in the studies we have identified. 

There is a clear need for consensus on which outcomes are important to people with Ménière’s disease, so that future studies can be designed with this in mind. Development of a core outcome set would be preferable as a guide for future trials. We understand that development of a core outcome set for Ménière's disease was underway, with a project registered on the COMET website (https://www.comet-initiative.org/Studies/Details/818), but we have been unable to identify any results of this project, or ascertain whether it is ongoing. If a core outcome set is developed, this should include details on the recommended methods used to measure outcomes, ensuring that these are validated, reliable tools. Monitoring and reporting of adverse effects should be considered a routine part of any study, and should always occur ‐ this is inconsistent at present. Agreement is also needed on the appropriate times at which outcomes should be measured to adequately assess the different interventions.

Any decisions about which outcomes to measure, how to measure them and when to measure them must be made with input from people with Ménière’s disease, to ensure that the outcomes reported by trialists (and future systematic reviews) are relevant to those with the disease. 

For those considering development of a core outcome set, we would highlight that the use of the dichotomous outcome 'improvement' or 'no improvement' of vertigo may cause difficulties when interpreting the results. Individuals with Ménière's disease typically experience fluctuations in disease severity over time. Furthermore, they may have enrolled in a clinical trial at a time when their symptoms were severe. Therefore there is likely to be a natural tendency to improve over time, even for those who do not receive an intervention. The high rate of improvement in those who receive no treatment means that smaller studies are likely to be underpowered to detect a true effect of treatment. Ideally, agreement should be reached on what constitutes a *meaningful improvement* in vertigo symptoms, rather than simply considering any improvement as a positive outcome. 

Trialists should also be clear about the treatments that participants received before entry to the trial, throughout the trial, and the need for additional treatment during the course of the trial. People with Ménière's disease need to be able to understand whether interventions work in all people with the disease, or whether they might work best during certain phases of the disease ‐ perhaps as a first‐line therapy, or for people in whom other treatments have failed. 

## History

Protocol first published: Issue 12, 2021

## Appendices

### Appendix 1. AAO‐HNS definition of Ménière's disease

Definite Ménière's disease:

- Two or more spontaneous episodes of vertigo, each lasting 20 minutes to 12 hours.
- Audiometrically documented low‐ to medium‐frequency sensorineural hearing loss in one ear, defining the affected ear on at least one occasion before, during or after one of the episodes of vertigo.
- Fluctuating aural symptoms (hearing, tinnitus or fullness) in the affected ear.
- Not better accounted for by another vestibular diagnosis.

Probable Ménière's disease: 

- Two or more spontaneous episodes of vertigo, each lasting 20 minutes to 24 hours.
- Fluctuating aural symptoms (hearing, tinnitus or fullness) in the affected ear.
- Not better accounted for by another vestibular diagnosis.

Taken from Lopez‐Escamez 2015.

### Appendix 2. Trustworthiness Screening Tool

This screening tool has been developed by Cochrane Pregnancy and Childbirth. It includes a set of predefined criteria to select studies that, based on available information, are deemed to be sufficiently trustworthy to be included in the analysis. These criteria are:

**Research governance**

- Are there any retraction notices or expressions of concern listed on the Retraction Watch Database relating to this study?
- Was the study prospectively registered (for those studies published after 2010)? If not, was there a plausible reason?
- When requested, did the trial authors provide/share the protocol and/or ethics approval letter?
- Did the trial authors engage in communication with the Cochrane Review authors within the agreed timelines?
- Did the trial authors provide IPD data upon request? If not, was there a plausible reason?

**Baseline characteristics**

- Is the study free from characteristics of the study participants that appear too similar (e.g. distribution of the mean (SD) excessively narrow or excessively wide, as noted by Carlisle 2017)?

**Feasibility**

- Is the study free from characteristics that could be implausible? (e.g. large numbers of women with a rare condition (such as severe cholestasis in pregnancy) recruited within 12 months);
- In cases with (close to) zero losses to follow‐up, is there a plausible explanation?

**Results**

- Is the study free from results that could be implausible? (e.g. massive risk reduction for main outcomes with small sample size)?
- Do the numbers randomised to each group suggest that adequate randomisation methods were used (e.g. is the study free from issues such as unexpectedly even numbers of women 'randomised' including a mismatch between the numbers and the methods, if the authors say 'no blocking was used' but still end up with equal numbers, or if the authors say they used 'blocks of 4' but the final numbers differ by 6)?

Studies assessed as being potentially 'high risk' will be not be included in the review. Where a study is classified as 'high risk' for one or more of the above criteria we will attempt to contact the study authors to address any possible lack of information/concerns. If adequate information remains unavailable, the study will remain in 'awaiting classification' and the reasons and communications with the author (or lack of) described in detail.

The process is described in full in Figure 2. 

### Appendix 3. Search strategy

This search strategy has been designed to identify all relevant studies for a suite of reviews on various interventions for Ménière's disease. 

**Table:**

**CENTRAL (CRS) **	**Cochrane ENT Register (CRS) **	**MEDLINE (Ovid) **
1 MESH DESCRIPTOR Endolymphatic Hydrops EXPLODE ALL AND CENTRAL:TARGET 2 meniere*):AB,EH,KW,KY,MC,MH,TI,TO AND CENTRAL:TARGET 3 (endolymphatic near hydrops):AB,EH,KW,KY,MC,MH,TI,TO AND CENTRAL:TARGET 4 (labyrinth* near hydrops):AB,EH,KW,KY,MC,MH,TI,TO AND CENTRAL:TARGET 5 (labyrinth* near syndrome):AB,EH,KW,KY,MC,MH,TI,TO AND CENTRAL:TARGET 6 (aural near vertigo):AB,EH,KW,KY,MC,MH,TI,TO AND CENTRAL:TARGET 7 (labyrinth* near vertigo):AB,EH,KW,KY,MC,MH,TI,TO AND CENTRAL:TARGET 8 (cochlea near hydrops):AB,EH,KW,KY,MC,MH,TI,TO AND CENTRAL:TARGET 9 (vestibular near hydrops):AB,EH,KW,KY,MC,MH,TI,TO AND CENTRAL:TARGET 10 #1 OR #2 OR #3 OR #4 OR #5 OR #6 OR #7 OR #8 OR #9 AND CENTRAL:TARGET	1 MESH DESCRIPTOR Endolymphatic Hydrops EXPLODE ALL AND INREGISTER 2 (meniere*):AB,EH,KW,KY,MC,MH,TI,TO AND INREGISTER 3 (endolymphatic near hydrops):AB,EH,KW,KY,MC,MH,TI,TO AND INREGISTER 4 (labyrinth* near hydrops):AB,EH,KW,KY,MC,MH,TI,TO AND INREGISTER 5 (labyrinth* near syndrome):AB,EH,KW,KY,MC,MH,TI,TO AND INREGISTER 6 (aural near vertigo):AB,EH,KW,KY,MC,MH,TI,TO AND INREGISTER 7 (labyrinth* near vertigo):AB,EH,KW,KY,MC,MH,TI,TO AND INREGISTER 8 (cochlea near hydrops):AB,EH,KW,KY,MC,MH,TI,TO AND INREGISTER 9 (vestibular near hydrops):AB,EH,KW,KY,MC,MH,TI,TO AND INREGISTER 10 #1 OR #2 OR #3 OR #4 OR #5 OR #6 OR #7 OR #8 OR #9 AND INREGISTER 11 INREGISTER 12 * AND CENTRAL:TARGET 13 #11 NOT #12 14 #10 AND #13	1 exp Endolymphatic Hydrops/ 2 meniere*.ab,ti. 3 (endolymphatic adj3 hydrops).ab,ti. 4 (labyrinth* adj3 hydrops).ab,ti. 5 (labyrinth* adj3 syndrome).ab,ti. 6 (aural adj3 vertigo).ab,ti. 7 (labyrinth* adj3 vertigo).ab,ti. 8 (cochlea adj3 hydrops).ab,ti. 9 (vestibular adj3 hydrops).ab,ti. 10 1 or 2 or 3 or 4 or 5 or 6 or 7 or 8 or 9 11 randomized controlled trial.pt. 12 controlled clinical trial.pt. 13 randomized.ab. 14 placebo.ab. 15 drug therapy.fs. 16 randomly.ab. 17 trial.ab. 18 groups.ab. 19 11 or 12 or 13 or 14 or 15 or 16 or 17 or 18 20 exp animals/ not humans.sh. 21 19 not 20 22 10 and 21
**Embase (Ovid) **	**Web of Science Core Collection (Web of Knowledge) **	**Trial Registries **
1 exp Meniere disease/ 2 meniere*.ab,ti. 3 (endolymphatic adj3 hydrops).ab,ti. 4 (labyrinth* adj3 hydrops).ab,ti. 5 (labyrinth* adj3 syndrome).ab,ti. 6 (aural adj3 vertigo).ab,ti. 7 (labyrinth* adj3 vertigo).ab,ti. 8 (cochlea adj3 hydrops).ab,ti. 9 (vestibular adj3 hydrops).ab,ti. 10 1 or 2 or 3 or 4 or 5 or 6 or 7 or 8 or 9 11 Randomized controlled trial/ 12 Controlled clinical study/ 13 Random$.ti,ab. 14 randomization/ 15 intermethod comparison/ 16 placebo.ti,ab. 17 (compare or compared or comparison).ti. 18 ((evaluated or evaluate or evaluating or assessed or assess) and (compare or compared or comparing or comparison)).ab. 19 (open adj label).ti,ab. 20 ((double or single or doubly or singly) adj (blind or blinded or blindly)).ti,ab. 21 double blind procedure/ 22 parallel group$1.ti,ab. 23 (crossover or cross over).ti,ab. 24 ((assign$ or match or matched or allocation) adj5 (alternate or group$1 or intervention$1 or patient$1 or subject$1 or participant$1)).ti,ab. 25 (assigned or allocated).ti,ab. 26 (controlled adj7 (study or design or trial)).ti,ab. 27 (volunteer or volunteers).ti,ab. 28 human experiment/ 29 trial.ti. 30 11 or 12 or 13 or 14 or 15 or 16 or 17 or 18 or 19 or 20 or 21 or 22 or 23 or 24 or 25 or 26 or 27 or 28 or 29 31 (random$ adj sampl$ adj7 ("cross section$" or questionnaire$1 or survey$ or database$1)).ti,ab. 32 comparative study/ or controlled study/ 33 randomi?ed controlled.ti,ab. 34 randomly assigned.ti,ab. 35 32 or 33 or 34 36 31 not 35 37 Cross‐sectional study/ 38 randomized controlled trial/ or controlled clinical study/ or controlled study/ 39 (randomi?ed controlled or control group$1).ti,ab. 40 38 or 39 41 37 not 40 42 (((case adj control$) and random$) not randomi?ed controlled).ti,ab. 43 (Systematic review not (trial or study)).ti. 44 (nonrandom$ not random$).ti,ab. 45 Random field$.ti,ab. 46 (random cluster adj3 sampl$).ti,ab. 47 (review.ab. and review.pt.) not trial.ti. 48 we searched.ab. 49 review.ti. or review.pt. 50 48 and 49 51 update review.ab. 52 (databases adj4 searched).ab. 53 (rat or rats or mouse or mice or swine or porcine or murine or sheep or lambs or pigs or piglets or rabbit or rabbits or cat or cats or dog or dogs or cattle or bovine or monkey or monkeys or trout or marmoset$1).ti. and animal experiment/ 54 36 or 41 or 42 or 43 or 44 or 45 or 46 or 47 or 50 or 51 or 52 55 30 not 54 56 10 and 55	# 12 #11 AND #10 Indexes=SCI‐EXPANDED, SSCI, A&HCI, CPCI‐S, CPCI‐SSH, BKCI‐S, BKCI‐SSH, ESCI, CCR‐EXPANDED, IC Timespan=All years # 11 TOPIC: ((randomised OR randomized OR randomisation OR randomisation OR placebo* OR (random* AND (allocat* OR assign*) ) OR (blind* AND (single OR double OR treble OR triple) ))) Indexes=SCI‐EXPANDED, SSCI, A&HCI, CPCI‐S, CPCI‐SSH, BKCI‐S, BKCI‐SSH, ESCI, CCR‐EXPANDED, IC Timespan=All years # 10 #9 OR #8 OR #7 OR #6 OR #5 OR #4 OR #3 OR #2 OR #1 Indexes=SCI‐EXPANDED, SSCI, A&HCI, CPCI‐S, CPCI‐SSH, BKCI‐S, BKCI‐SSH, ESCI, CCR‐EXPANDED, IC Timespan=All years # 9 TOPIC: (vestibular NEAR/3 hydrops) Indexes=SCI‐EXPANDED, SSCI, A&HCI, CPCI‐S, CPCI‐SSH, BKCI‐S, BKCI‐SSH, ESCI, CCR‐EXPANDED, IC Timespan=All years # 8 TOPIC: (cochlea NEAR/3 hydrops) Indexes=SCI‐EXPANDED, SSCI, A&HCI, CPCI‐S, CPCI‐SSH, BKCI‐S, BKCI‐SSH, ESCI, CCR‐EXPANDED, IC Timespan=All years # 7 TOPIC: (labyrinth* NEAR/3 vertigo) Indexes=SCI‐EXPANDED, SSCI, A&HCI, CPCI‐S, CPCI‐SSH, BKCI‐S, BKCI‐SSH, ESCI, CCR‐EXPANDED, IC Timespan=All years # 6 TOPIC: (labyrinth* adj3 vertigo) Indexes=SCI‐EXPANDED, SSCI, A&HCI, CPCI‐S, CPCI‐SSH, BKCI‐S, BKCI‐SSH, ESCI, CCR‐EXPANDED, IC Timespan=All years # 5 TOPIC: (aural NEAR/3 vertigo) Indexes=SCI‐EXPANDED, SSCI, A&HCI, CPCI‐S, CPCI‐SSH, BKCI‐S, BKCI‐SSH, ESCI, CCR‐EXPANDED, IC Timespan=All years # 4 TOPIC: (labyrinth* NEAR/3 syndrome) Indexes=SCI‐EXPANDED, SSCI, A&HCI, CPCI‐S, CPCI‐SSH, BKCI‐S, BKCI‐SSH, ESCI, CCR‐EXPANDED, IC Timespan=All years # 3 TOPIC: (labyrinth* NEAR/3 hydrops) Indexes=SCI‐EXPANDED, SSCI, A&HCI, CPCI‐S, CPCI‐SSH, BKCI‐S, BKCI‐SSH, ESCI, CCR‐EXPANDED, IC Timespan=All years # 2 TOPIC: (endolymphatic NEAR/3 hydrops) Indexes=SCI‐EXPANDED, SSCI, A&HCI, CPCI‐S, CPCI‐SSH, BKCI‐S, BKCI‐SSH, ESCI, CCR‐EXPANDED, IC Timespan=All years # 1 TOPIC: (meniere*) Indexes=SCI‐EXPANDED, SSCI, A&HCI, CPCI‐S, CPCI‐SSH, BKCI‐S, BKCI‐SSH, ESCI, CCR‐EXPANDED, IC Timespan=All years	Clinicaltrials.gov menieres or meniere or meniere's | Interventional Studies ICTRP Meniere*

The date restrictions applied to the September 2022 update searches were as follows:

**Table:**

** **	**September 2022 update**
**CENTRAL**	15/09/2021_TO_14/09/2022:CRSINCENTRAL AND CENTRAL:TARGET
**ENT register**	No new records added to register since search was run
**MEDLINE**	23 limit 22 to ed=20210915‐20220914 24 limit 22 to dt=20210915‐20220914 25 23 or 24
**Embase**	57 limit 56 to dd=20210915‐20220914
**Web of Science**	Timespan: 2021‐09‐15 to 2022‐09‐14 (Index Date)
**Clinicaltrials.gov**	First posted from 09/15/2021 to 09/14/2022
**ICTRP**	Date of registration after 15/09/2021
**Google Scholar**	Year: 2021 or 2022

## Data and analyses

**Comparison 1:** Positive pressure therapy compared to placebo

Outcome or subgroup title	No. of studies	No. of participants	Statistical method	Effect size
1.1 Improvement in vertigo frequency (3 to < 6 months)	1	77	Risk Ratio (M‐H, Random, 95% CI)	1.19 [0.82, 1.71]
1.2 Change in vertigo: global score (3 to < 6 months)	1	68	Mean Difference (IV, Random, 95% CI)	‐5.31 [‐11.67, 1.05]
1.3 Change in vertigo: frequency of vertigo (3 to < 6 months)	3	202	Mean Difference (IV, Random, 95% CI)	‐0.84 [‐2.12, 0.45]
1.4 Disease‐specific health‐related quality of life (3 to < 6 months)	1	77	Mean Difference (IV, Random, 95% CI)	0.00 [‐0.10, 0.10]
1.5 Change in hearing (3 to < 6 months)	2	123	Mean Difference (IV, Random, 95% CI)	2.49 [‐6.17, 11.14]

## Characteristics of studies

### Characteristics of included studies [ordered by study ID]

**Table:** Gates 2004

***Study characteristics***		
Methods	Parallel‐group, double‐blind, randomised controlled trial with 4 months total duration of treatment and follow‐up	
Participants	Participants had a clinical diagnosis of active, definite, unilateral cochleovestibular Ménière's disease, causing disruptive levels of vertigo (at least 2 definitive attacks per month for the 2 months prior to entering the study) despite at least 3 months of treatment with a low‐sodium diet, with or without diuretics **Setting:** Multicentre trial conducted at 4 study sites in the USA from February 2002 until April 2003 **Sample size:** **Number randomised:** 67 **Number completed:** 62 Note that an additional 5 participants declared themselves to be 'treatment failures' at the 2‐month follow‐up, and did not attend the 4‐month follow‐up. However, their 2‐month data were also included as 4‐month data in the analysis. **Participant baseline characteristics** **Age: ** Meniett group mean 49.7 years (SD 9.0), range 34 to 67 Control group mean 48.8 years (SD 9.1) range 36 to 71 **Gender:** Meniett group 10 (34%) male: 20 (67%) female Control group 10 (31%) male: 22 (69%) female **Probable/definite Ménière's disease:** All participants had definite disease **Duration of disease:** Not reported for individual groups. Overall, duration of symptoms was reported to be 4.5 years (IQR 2 to 7 years) **Attack frequency at baseline:** Meniett group mean proportion of days with definitive vertigo 0.20 (SD 0.27) Control group mean proportion of days with definitive vertigo 0.24 (SD 0.22) **Hearing loss at baseline:** Assessed with PTA, using the average of 0.25 kHz, 0.50 kHz and 1.0 kHz in the affected ear Meniett group mean 56.1 dB HL (SD 19.7) Control group mean 51.5 dB HL (SD 18.7) **Measure of tinnitus at baseline:** Not reported **Number of participants with bilateral disease:** All participants had unilateral disease **Inclusion criteria:** Active, unilateral, definite Ménière's disease according to the AAO‐HNS 1995 criteria At least 2 attacks per month for the 2 months prior to study entry Insufficient relief from a low‐sodium diet, with or without diuretics Documented low‐frequency sensorineural hearing loss and a history of fluctuating hearing Functionality level of 2 to 4 Normal auditory brainstem responses An abnormal electrocochleogram in the affected ear, i.e. SP/AP click ratio of greater than 0.39 or a toneburst SP of 2.0 μV or larger **Exclusion criteria:** None reported	
Interventions	**Intervention (n = 34 randomised, n = 30 completed)** Meniett device All participants had a tympanostomy tube fitted. The Meniett device was self‐administered 3 times daily. The pressure‐generator was used to deliver 0.6 second pressure pulses at 6 Hz within the range of 0 cm to 20 cm H_2_O) to the ear canal through a polyethylene tube with a close‐fitting cuff. The 5‐minute treatment sequence had 3 cycles, each with 1 minute of pressure pulses and 40 seconds of pause. The device was used for 4 months in total. **Comparator (n = 33 randomised, n = 32 completed)** Placebo device All participants had a tympanostomy tube fitted. The device and usage was identical to the Meniett device, but did not generate pressure pulses. **Background interventions administered to all participants** Participants were instructed to take a 1500 mg/day sodium diet to optimise symptom control. Dietary compliance was not monitored. Participants were allowed to continue taking pre‐study medications as needed. All participants had a tympanostomy tube fitted.	
Outcomes	**Primary outcomes relevant to this review:** **Improvement in vertigo** No data reported **Change in vertigo** Global symptoms score. Participants rated their vertigo daily using a symptom card, which included the duration and severity of symptoms. 0 = Vertigo‐free day 1 = Day with a mild attack 2 = Day with a moderately severe attack lasting more than 20 minutes 3 = Day with a severe attack lasting an hour or more or accompanied by nausea or vomiting 4 = The worst attack ever experienced to date A definitive vertigo day was any day with a vertigo score of 2, 3 or 4. Total symptom score in a given 4‐week period will include duration, severity and frequency of attacks. Authors report total proportion of days with "definitive vertigo" ‐ classed as a score of ≥ 2. Therefore, although a global score was used, only the frequency of episodes was reported. **Serious adverse events** Authors do comment on adverse effects, but it is not clear if these were systematically assessed and reported **Secondary outcomes relevant to this review:** **Disease‐specific health‐related quality of life** Not reported **Hearing** Assessed using a pure tone average of 3 low‐tone frequencies (0.25 kHz, 0.5 kHz and 1 kHz) **Tinnitus ** Not reported **Other adverse effects** Authors do comment on adverse effects, but it is not clear if these were systematically assessed and reported **Other outcomes reported in the study:** Total number of definitive vertigo days for the entire group (not mean scores, but summed for the group) at each month Proportion of days with definitive vertigo across all 4 months (as opposed to at 4 months of follow‐up) Proportion of sick days	
Funding sources	Funding was provided by Medtronic Xomed as an unrestricted grant to each of the 4 study centres	
Declarations of interest	Dr. Gates served as a paid consultant to Medtronic Xomed at a scientific retreat in 2002. No other declarations are stated.	
Notes	**Research integrity checklist:** No retractions/expressions of concern were noted regarding this study Publication was prior to 2010; no protocol was identified No concerns regarding baseline characteristics of trial participants No concerns regarding loss to follow‐up No concerns regarding plausibility of results Block randomisation was used to ensure balanced allocation to the 2 groups	
***Risk of bias***		
**Bias**	**Authors' judgement**	**Support for judgement**
Random sequence generation (selection bias)	Low risk	Comment: although details are not explicit, authors state that a "randomised block design (balanced for every 4 subjects)" was used, stratified for gender and normal/abnormal caloric test results. This appears to have been generated centrally, and then contact was made by individual study sites when patients were ready for randomisation. We considered it likely that the method for randomisation was adequate.
Allocation concealment (selection bias)	Low risk	Quote: "The Meniett manager at each site received the assignment from the study monitor and both recorded the coded treatment assignment and device serial number". Comment: group allocation appears to have been produced centrally by a third party, therefore participants and personnel at individual study sites would be unable to predict group allocation.
Blinding of participants and personnel (performance bias) All outcomes	Low risk	Quote: "Participants and evaluators were blinded to the treatment assignment. The active and inactive devices were identical in appearance and both generated a similar clicking sound and light display during operation. The devices were sealed to assure integrity". "Reasonable attempts were made to maintain participant blinding by having sealed devices with identical appearance and acoustic properties. Whether the participants might have tested the device in some way to determine its activity is unknown. Many participants were unaware of the presence or absence of pressure sensations during device use and were unable to hear the acoustic signals during device operation because of their hearing loss. Therefore, we believe the blinding was effective and that participant equipoise was maintained". Comment: we considered it likely that participants and personnel were likely to be sufficiently blinded to the intervention.
Blinding of outcome assessment (detection bias) All outcomes	Low risk	Quote: "Participants and evaluators were blinded to the treatment assignment. The active and inactive devices were identical in appearance and both generated a similar clicking sound and light display during operation. The devices were sealed to assure integrity" Comment: primary outcomes were assessed by blinded participants. Additional outcomes were stated to be assessed by blinded evaluators.
Incomplete outcome data (attrition bias) All outcomes	High risk	Comment: participants were able to self‐declare "treatment failure" at 2 months, and were then excluded from 4‐month follow‐up. This occurred at a differential rate in the 2 groups (n = 4 control, n = 1 treatment). Results may therefore have been influenced by true outcome. Bias may have occurred because of the possibility of spontaneous resolution of symptoms by the 4 month time‐point.
Selective reporting (reporting bias)	High risk	Comment: no registered protocol therefore unable to assess fully. Methods state that vertigo was summarised in 2 ways ‐ severity (monthly total of counting vertigo scores) and frequency ‐ the proportion of counting vertigo days per time period. However, monthly total scores are only reported for the whole group (i.e. the sum of episodes in the entire group), not for individual participants, therefore we are unable to ascertain the mean monthly severity score. It is unclear whether changes in the entire group are informative.
Other bias	High risk	Comment: change in vertigo is reported using an unvalidated scoring system.

**Table:** Gurkov 2012

***Study characteristics***		
Methods	Double‐blind, single‐centre, parallel‐group RCT with 4 months duration of treatment and follow‐up	
Participants	**Setting:** Conducted at 2 outpatient facilities of an otolaryngology department in a tertiary referral centre in Germany. Study took place from November 2004 until November 2008. **Sample size:** **Number randomised:** 74 participants **Number completed:** 68 participants **Participant baseline characteristics** **Age:** Positive pressure group: mean 57 years, range 24 to 85 years Control group: mean 52 years, range 19 to 74 years **Gender:** Positive pressure group: 19 males (50%): 19 females (50%) Control group: 19 males (53%): 17 females (47%) **Probable/definite Ménière's disease:** All definite Ménière's disease (inclusion criterion) **Duration of disease:** Positive pressure group: mean 43 months, range 5 to 186 months Control group: mean 57 months, range 4 to 276 months **Attack frequency at baseline:** Positive pressure group: mean number of definitive vertigo days per 4‐week period at baseline = 6.5 Control group: mean number of definitive vertigo days per 4‐week period at baseline = 5.94 **Hearing loss at baseline:** Positive pressure group: mean hearing threshold 49.15 dB HL (measured with pure‐tone audiogram at 0.25 kHz, 0.5 kHz and 1 kHz) Control group: mean hearing threshold 41.66 dB HL (measured with pure‐tone audiogram at 0.25 kHz, 0.5 kHz and 1kHz) **Measure of tinnitus at baseline:** Not reported **Number of participants with bilateral disease:** None (inclusion criterion: unilateral disease) **Inclusion criteria:** Definite Ménière's disease as per the diagnostic criteria of the AAO‐HNS 1995. Two or more vertigo attacks (lasting at least 20 minutes) per month in the last 2 months. Treatment with betahistine (from 16 mg to 24 mg 3 times daily for 3 months) without subjective vertigo control. **Exclusion criteria:** Bilateral Ménière's disease. Previous destructive or surgical therapy (e.g. gentamicin or endolymphatic sac surgery). Aged less than 18 years.	
Interventions	**Intervention (n = 38 randomised, n = 37 completed)** Meniett device. Participants underwent 16 weeks of low‐pressure therapy using the Meniett device. Pulses were delivered at 6 Hz for 0.6 seconds within the range of 0 to 20 cm H_2_O via a polyethylene tube with a close fitting cuff. The treatment was for 5 minutes, and had 3 cycles, each with 1 minute of pressure pulses and 40 seconds of no pressure. The device was used 3 times daily. **Comparator (n = 36 randomised, n = 31 completed)** Placebo device. The device was used in the same way as the active device, and had identical properties, but only generated a slight pressure increase to 2 cm H_2_O. The authors state that it is unlikely that this would have any effect, but they cannot be sure. **Background interventions administered to all participants** All participants were administered with a ventilation tube as this is required to deliver the intervention. All participants were advised to continue on betahistine depending on pre‐treatment medication (i.e. from 16 mg to 24 mg 3 times daily).	
Outcomes	**Primary outcomes relevant to this review:** **Improvement in vertigo** Not reported **Change in vertigo** Change in vertigo was assessed according to a 'standardised format' in analogy to that used in Gates 2004. This is a self‐reported symptom diary, where participants record the severity of vertigo and its influence on daily activities using a 5‐point Likert scale (0 to 4). **Serious adverse events** Narrative summary only, which states that "no adverse effects of Meniett device treatment were reported". It is unclear whether these were systematically assessed. **Secondary outcomes relevant to this review:** **Disease‐specific health‐related quality of life** Not reported **Hearing** Assessed using pure tone audiometry at 3 frequencies (0.25 kHz, 0.5 kHz and 1 kHz) **Tinnitus ** Not reported **Other adverse effects** Narrative summary only, which states that "no adverse effects of Meniett device treatment were reported". It is unclear whether these were systematically assessed. **Other outcomes reported in the study:** Vertigo‐free days Activity score Caloric stimulus induced slow‐phase nystagmus velocity	
Funding sources	The Meniett devices were provided by Medtronic Xomed for the duration of the study	
Declarations of interest	The authors declare no conflicts of interest	
Notes	**Research integrity checklist:** No retractions/expressions of concern were identified No prospective trial registration was identified (retrospective registration with clinicaltrials.gov) Baseline characteristics of the 2 groups do not appear excessively similar Plausible loss to follow‐up was reported over the course of the trial No implausible results were identified No concern over randomisation	
***Risk of bias***		
**Bias**	**Authors' judgement**	**Support for judgement**
Random sequence generation (selection bias)	Unclear risk	Quote: "patients [...] were individually randomised into either the active treatment group or the placebo group". Comment: no further details on randomisation process.
Allocation concealment (selection bias)	Unclear risk	Quote: "patients [...] were individually randomised into either the active treatment group or the placebo group". Comment: no further details on allocation process. Unclear whether group allocation could have been foreseen.
Blinding of participants and personnel (performance bias) All outcomes	Low risk	Quote: "Both physicians and patients were blinded to the treatment allocation until the end of the treatment period." Comment: placebo device used, identical in appearance. We assume that the small pressure generated by the placebo device would have been indistinguishable to participants from the active device (and therefore that blinding was adequate).
Blinding of outcome assessment (detection bias) All outcomes	Low risk	Quote: "The placebo device had identical acoustic properties to the Meniett® device, but only produced a slight pressure increase, to 2 cm H_2_O." Comment: the outcome assessors were the participants themselves. The trialists made attempts to reduce the risk of the participants knowing if they were on the treatment or control arm by ensuring the placebo device was as indistinguishable as possible from the Meniett device. This does, however, raise the question of whether there was some degree of therapeutic effect from the placebo device. Overall, we considered blinding to be adequate, therefore the study is at low risk of performance bias.
Incomplete outcome data (attrition bias) All outcomes	High risk	Comment: dropouts in placebo arm almost 14%, compared to 2% in the active treatment arm. One dropout due to "non‐compliance" indicating that intention‐to‐treat analysis was not used. Differential dropout due to treatment failure in the placebo arm, suggestive that the reason for missing outcome data is likely to be related to true outcome.
Selective reporting (reporting bias)	Unclear risk	Comment: no prospectively registered trial protocol was available to assess this domain.
Other bias	High risk	Comment: the use of a placebo device that generated a small pressure may have caused bias in the overall estimated effect. An unvalidated scoring system was used to assess the change in vertigo during the trial.

**Table:** Russo 2017

***Study characteristics***		
Methods	Parallel‐group, double‐blind, placebo‐controlled RCT with 6 weeks duration of treatment and further 6 weeks follow‐up (total study duration 12 weeks)	
Participants	**Setting:** Multicentre study conducted across 17 ENT centres in France. Study dates are not reported. **Sample size:** **Number randomised:** 97 participants **Number completed:** 77 participants **Participant baseline characteristics** **Age:** Positive pressure group: mean 52 years (standard error 1.6 years) Control group: mean 50 years (standard error 1.9 years) **Gender:** Positive pressure group: 20 males (40%): 29 females (60%) Control group: 23 males (47%): 25 females (53%) **Probable/definite Ménière's disease:** All participants had definite disease (inclusion criterion) **Duration of disease:** Positive pressure group: mean 7 months (SE 0.97 months), range 0.15 to 26.5 months Control group: mean 5 months (SE 0.81 months), range 0.22 to 24.5 months **Attack frequency at baseline:** Positive pressure group: mean 3.2 (SE 0.4) episodes lasting > 20 minutes during 3 weeks of wash‐out phase Control group: mean 4.3 (SE 0.6) episodes lasting > 20 minutes during 3 weeks of wash‐out phase **Hearing loss at baseline:** Not reported **Measure of tinnitus at baseline:** Not reported **Number of participants with bilateral disease:** None; all participants had unilateral disease (inclusion criterion) **Inclusion criteria:** Adults over 18 years old age. Affected by a stage 2 or greater unilateral, definite Ménière's disease, according to the AAO‐HNS 1995 criteria. Patients were included if they experienced at least 2 episodes of rotatory vertigo in the preceding 2 months (vertigo lasting at least 20 minutes, with a free interval of 12 hours), with or without associated tinnitus, and/or a sensation of fullness in the ear. The impact of vertigo on the patient’s daily life had to be at level 3 or greater on the functionality level according to AAO‐HNS 1995. **Exclusion criteria:** Previous surgical treatment or chemical labyrinthectomy for Ménière's disease	
Interventions	**Intervention (n = 49 randomised, n = 41 completed)** Meniett device. A ventilation tube was inserted. Participants used the Meniett device (which generates pulse pressure to the ear) 3 times daily, for 15 minutes each time. Pressure pulsed waves were at a frequency of 6 Hz and a maximum pressure of 12 cm/H_2_O. The device was used for 6 weeks, then discontinued, and participants were followed up for a further 6 weeks. **Comparator (n = 48 randomised, n = 36 completed)** Placebo group. A ventilation tube was inserted. A placebo device ‐ identical in all aspects to the active device ‐ was used, which did not generate pulse pressures. Use and follow‐up was the same as for the active group. **Background interventions administered to all participants** Both groups had a ventilation tube inserted. A wash‐out period of 8 weeks was included, suggesting that participants had to discontinue any other medication prior to entry to the trial.	
Outcomes	**Primary outcomes relevant to this review:** **Improvement in vertigo** Reported as the number of participants in whom the frequency of vertigo episodes (lasting > 20 minutes) at the end of treatment was lower than that at the start of treatment. Vertigo was rated as worsened, no change or improved. It appears that this rating was made by the study personnel, based on the change in frequency of episodes. It was not a subjective report of global improvement by the participants themselves. **Change in vertigo** Assessed as the number of vertigo episodes lasting > 20 minutes over a 3‐week period **Serious adverse events** Not assessed or reported **Secondary outcomes relevant to this review:** **Disease‐specific health‐related quality of life** Apparently assessed using the AAO‐HNS 1995 FLS (range 1 to 6, higher scores = worse quality of life). However, we note that the footnote to Table IV states that these data are according to a visual analogue scale. **Hearing** Not assessed or reported **Tinnitus ** Not assessed or reported **Other adverse effects** Not assessed or reported **Other outcomes reported in the study:** Number of short duration vertigo episodes (5 to 20 minutes), per 21‐day period Number of "dizziness episodes", identified as vertigo episodes lasting < 5 minutes, per 21‐day period	
Funding sources	Medtronic Xomed, manufacturer of Meniett device	
Declarations of interest	Quote: "This trial was partially financed by Medtronic Xomed, Inc. (Jacksonville, FL). The authors have no other funding, financial relationships, or conflicts of interest to disclose"	
Notes	**Research integrity checklist:** No retractions/expressions of concern No trial registration or protocol was available Baseline characteristics of participants are not excessively similar Plausible loss to follow‐up was reported No implausible results are noted Blocked randomisation was used, which accounts for similar allocation to each group	
***Risk of bias***		
**Bias**	**Authors' judgement**	**Support for judgement**
Random sequence generation (selection bias)	Low risk	Quote: "Randomization was performed by blocks of four. Each center received a block of four devices (2 Meniett [Medtronic Xomed] and 2 placebo). If necessary, according to the rate of inclusion in a given center, more blocks of four devices were distributed to the center. The boxes were randomly numbered and the physician did not know their content and had to distribute them to the patients." Comment: central randomisation, separate to the individual recruiting centres.
Allocation concealment (selection bias)	Low risk	Quote: "The boxes were randomly numbered and the physician did not know their content and had to distribute them to the patients." Comment: central allocation by a third party. Devices were also identical in appearance, therefore even opening boxes would not have indicated which group the participant would be allocated to.
Blinding of participants and personnel (performance bias) All outcomes	Low risk	Quote: "The boxes were randomly numbered and the physician did not know their content and had to distribute them to the patients. The placebo was identical in all aspects to the active device but did not generate pressure pulses." Comment: we assume that participants were unable to detect the pressure pulses, therefore consider blinding to be adequate.
Blinding of outcome assessment (detection bias) All outcomes	Low risk	Comment: outcomes were reported by the (blinded) participants themselves.
Incomplete outcome data (attrition bias) All outcomes	High risk	Comment: differential dropout between the 2 groups, with 25% loss in the placebo arm, and 16% loss in the Meniett group. Reasons for dropout are not clear, but could potentially be related to the efficacy of the interventions, and the difference is large enough to impact the outcome of the number of people who had improved.
Selective reporting (reporting bias)	High risk	Comment: data on severity of vertigo episodes were collected but are not reported.
Other bias	Low risk	Comment: no other concerns identified.

AAO‐HNS: American Academy of Otolaryngology ‐ Head and Neck Surgery; AP: action potential; dB decibels; ENT: ear, nose and throat; FLS Functional Level Scale; HL: hearing loss; IQR: interquartile range; kHz: kilohertz; PTA: pure tone average; SD: standard deviation; SE: standard error; SP: summating potential

### Characteristics of excluded studies [ordered by study ID]

**Table:**

Study	Reason for exclusion
Barbara 2001	This is not an RCT, it is a prospective cohort study
Conde 1965	This is a review article on the treatment of vertigo, not an RCT
Densert 1997	This RCT followed up patients immediately after exposure to positive pressure therapy, therefore the duration of follow‐up was insufficient for this review (< 3 months)
Odkvist 2000	This RCT only followed participants up for 2 weeks, therefore the duration of follow‐up was insufficient
Odkvist 2004	This conference abstract is a summary of other trials, and does not report any primary data
Richards 1971	This is a narrative review article and not an RCT
Thomsen 2005	This RCT only followed participants up for a maximum of 8 weeks, therefore the follow‐up period was insufficient for this review
UMIN000002356	This is a single‐arm study, not an RCT
UMIN000002357	This is a single‐arm study, not an RCT
UMIN000005544	This is a single‐arm study, not an RCT
UMIN000007659	This is a single‐arm study, not an RCT
UMIN000017710	This is a single‐arm study, not an RCT

RCT: randomised controlled trial

### Characteristics of studies awaiting classification [ordered by study ID]

**Table:** Bouccara 2005

Methods	—
Participants	—
Interventions	—
Outcomes	—
Notes	We have been unable to locate a copy of the conference proceedings in which this abstract was published.

**Table:** Feijen 2005

Methods	—
Participants	—
Interventions	—
Outcomes	—
Notes	We have been unable to locate a copy of the conference proceedings in which this abstract was published.

**Table:** Gates 2003

Methods	—
Participants	—
Interventions	—
Outcomes	—
Notes	We have been unable to locate a copy of this conference abstract.

**Table:** Gates 2005

Methods	—
Participants	—
Interventions	—
Outcomes	—
Notes	We have been unable to locate a copy of the conference proceedings in which this abstract was published.

## Differences between protocol and review

Due to the paucity of data for most outcomes, we were unable to carry out a number of pre‐planned sensitivity analyses and subgroup analyses. We planned to use the Trustworthiness Screening Tool from Cochrane Pregnancy and Childbirth to identify studies for inclusion in the main analysis. However, as described in Selection of studies, we did not exclude studies from the main analysis on the basis of concerns whilst using this tool. We considered that the overall certainty of the review findings (all very low‐ or low‐certainty) would not be impacted by this decision. 
